# Development of a highly sensitive magneto-enzyme lateral flow immunoassay for dengue NS1 detection

**DOI:** 10.7717/peerj.7779

**Published:** 2019-09-25

**Authors:** Tien V. Tran, Ba V. Nguyen, Thao T.P. Nguyen, Tung T. Tran, Khanh G. Pham, Quang B. Le, Binh N. Do, Hung N. Pham, Chuyen V. Nguyen, Duong P.H. Dinh, Van T. Ha, Trang H.T. Doan, Hoa Q. Le

**Affiliations:** 1 Military Medical University, Hanoi, Vietnam; 2 School of Biotechnology and Food Technology, Hanoi University of Science and Technology, Hanoi, Vietnam; 3 Nguyen Hue High School for Gifted Students, Hanoi, Vietnam; 4 Hanoi-Amsterdam High School for Gifted Students, Hanoi, Vietnam

**Keywords:** Dengue virus, NS1 protein, Magnetic lateral flow immunoassay, Signal amplification, Rapid method

## Abstract

**Background:**

Dengue infection represents a global health issue of growing importance. Dengue non-structural protein 1 (NS1) plays a central role in the early detection of the disease. The most common method for NS1 detection is testing by lateral flow immunoassays (LFIAs) with varying sensitivity. In this study, we present a highly sensitive magneto-enzyme LFIA for prompt diagnosis of dengue.

**Methods:**

We have demonstrated the development of a magneto-enzyme LFIA combining super-paramagnetic nanoparticles as labels and Biotin–Streptavidin signal amplification strategy to detect dengue NS1. Factors affecting the test performance including antibody pair, super-paramagnetic nanoparticle size, nitrocellulose membrane type, amounts of detection and capture antibodies, and amounts of Streptavidin-polyHRP were optimized. Analytical sensitivity and cross-reactivity were determined. Clinical performance of the novel assay was evaluated using a panel of 120 clinical sera.

**Results:**

This newly developed assay could detect NS1 of all four serotypes of dengue virus (DENV). The limit of detection (LOD) was found to be as low as 0.25 ng ml^−1^ for DENV-1 and DENV-3, 0.1 ng ml^−1^ for DENV-2, and 1.0 ng ml^−1^ for DENV-4. The LOD for DENV-2 was a 50-fold improvement over the best values previously reported. There was an absence of cross-reactivity with Zika NS1, Hepatitis B virus, Hepatitis C virus, and Japanese encephalitis virus. The sensitivity and specificity of the novel assay were 100% when tested on clinical samples.

**Conclusions:**

We have successfully developed a magneto-enzyme LFIA, allowing rapid and highly sensitive detection of dengue NS1, which is essential for proper management of patients infected with DENV.

## Introduction

Dengue is a mosquito-borne disease caused by the dengue virus (DENV) of the family *Flaviviridae*. Although it has existed for centuries, dengue remains a global health issue with 3.6 billion people living with the risk of infection. An estimated 390 million dengue cases occur annually in tropical and subtropical regions such as South-East Asia, Western Pacific, and Africa (Bhatt et al., 2013; Brady et al., 2012; World Health Organization (WHO), 2019).

Dengue is associated with symptoms of different severity, lasting from 3 days to 1 week. Severe dengue patients potentially suffer bleeding, low platelet level or plasma leakage, leading to dengue shock syndrome or death (Kularatne, 2015). Therefore, early and accurate diagnosis is critical for proper medical care and lowering the risk of severe complications. Presently, detection of non-structural protein 1 (NS1) antigen is a common approach for early diagnosis of dengue (Chaterji et al., 2011; Najioullah et al., 2011). Aside from being present in both primary and secondary infections, NS1 is detectable in the blood of patients from the first day of symptom onset, up to day 9 or early convalescence (Alcon et al., 2002; Young et al., 2000). NS1 could be detected by commercial enzyme-linked immunosorbent assay (ELISA) or lateral flow immunoassay (LFIA). In comparison to ELISA, LFIA displays several advantages, such as low cost, rapid, and simple operation, allowing point-of-care testing of dengue. However, several studies have pointed out that the sensitivities of commercial NS1 LFIAs are variable between investigations (Blacksell et al., 2011; Lima et al., 2010; Osorio et al., 2010; Pok et al., 2010; Zainah et al., 2009). A meta-analysis by Zhang et al. (2014) revealed that sensitivities of commercial LFIAs ranged from 48% (95% CI [38–59%]) to 90% (95% CI [87–93%]).

Several strategies could be employed to enhance the sensitivity of LFIAs. For instance, the selection of antibody pairs, silver enhancement, using magnetic nanoparticles as the labeling material or signal amplification (Zherdev & Dzantiev, 2018). Magnetic nanoparticles represent an attractive labeling material in LFIA (Huang et al., 2018; Linares et al., 2012; Wu et al., 2017). Signals generated by this material in the entire volume of the nitrocellulose membrane could be quantified by a Magnetic Reader (Wang et al., 2009), thus, potentially increasing the analytical sensitivity of the assay. Alternatively, a signal amplification step could be performed, allowing the observation of the results with the naked eye (Cho & Irudayaraj, 2013). Combining the use of magnetic nanoparticles and a signal amplification step by horseradish peroxidase (HRP) allowed detecting directly *Listeria monocytogenes* within 2 h with the limit of detection (LOD) down to 95 CFU ml^−1^ (Cho & Irudayaraj, 2013). In the present study, based on the same approach, we have developed a magneto-enzyme LFIA for detection of dengue NS1 with enhanced sensitivity.

## Materials and Methods

### Clinical samples and ethics statement

A panel of 120 sera was used to evaluate the clinical performance of the magneto-enzyme LFIA. The sera were obtained within 9 days post-onset of illness at Vietnam Military Medical University (Hanoi, Vietnam). All samples were tested for dengue RNA by RT-qPCR as previously described (Gurukumar et al., 2009) and also for dengue-specific IgM antibodies by Dengue IgG/IgM 3.0 Combo rapid test (CTK Biotech, Inc., Poway, CA, USA). Typing of positive samples was performed by nested RT-PCR (Lanciotti et al., 1992). Clinical samples positive for Hepatitis B virus (HBV) (viral load = 2.2 × 10^5^ IU ml^−1^), Hepatitis C virus (HCV) (viral load = 4.3 × 10^4^ IU ml^−1^), and Japanese encephalitis virus (positive with DRG^®^ JE IgM Antibody Capture ELISA) were also collected to determine the cross-reactivity of the assay. All samples were stored at −80 °C until use.

This study was approved by the Research Ethics Committee of Vietnam Military Medical University, Approval No. 18/QD-HDDD. Written informed consent was obtained from each participant or their legal guardians. Patients’ anonymity and confidentiality were guaranteed by the researchers involved in the study.

### Preparation of biotinylated, antibody-conjugated magnetic nanoparticles

Conjugation of the Carboxyl-Adembeads (with a diameter of 100, 200, or 300 nm; Ademtech, Pessac, France) with monoclonal antibodies (10-2699 from Fitzgerald, North Acton, MA, USA or HM164 from EastCoast Bio, North Berwick, ME, USA; mAb) was performed as per manufacturer’s specifications with some modifications. To form mAb-Adembead complexes, 100 µl (three mg) of Carboxyl-Adembeads was first activated by incubating, on a Dynal Biotech rotary shaker, Thermo Fisher Scientific, Dynal Biotech, Waltham, MA, USA (15 rpm), with 240 µl of 1-ethyl-3-(3-dimethylaminopropyl) carbodiimide (EDC) (Sigma, St. Louis, MO, USA) at the concentration of 10 mg ml^−1^. After activation step, excess of EDC was removed and the nanoparticles were washed with one ml of Activation Buffer 1× (#10101; Ademtech, Pessac, France). Conjugation was then carried out by incubating the nanoparticles with 150 µg of detection antibody at room temperature for 2 h on a Dynal Biotech rotary shaker (15 rpm). Blocking of free carboxyl groups on magnetic nanoparticles was performed by incubating the immunocomplexes with 600 µl of bovine serum albumin (Sigma, St. Louis, MO, USA) (BSA, 0.5 mg ml^−1^) at 37 °C for 30 min. After being washed with PBS 1×, primary amine groups of BSA and mAbs on the immunocomplexes were biotinylated using EZ-Link™ Sulfo-NHS-Biotin (Thermo Fisher Scientific, Waltham, MA, USA) as per manufacturer’s instructions. Specifically, the complexes were dissolved in 300 µl PBS 1× (pH = 7.4) before being mixed with 15 µl of 10 mM Sulfo-NHS-Biotin. The reaction mixture was incubated on a Dynal Biotech rotary shaker at 15 rpm for 2 h at room temperature before being washed with Storage buffer 1× (#10201; Ademtech, Pessac, France). The final product (biotinylated, mAb-conjugated magnetic beads) was stored in 1,500 µl of Storage buffer 1× (#10201; Ademtech, Pessac, France).

### Preparation of the immunochromatography strips

Test strips were manufactured according to Posthuma-Trumpie, Korf & Van Amerongen (2008). Briefly, a Linomat V (Camag, Muttenz, Switzerland) was used to dispense antibodies onto a nitrocellulose membrane of 2.5 cm wide. For the control line, goat anti-mouse IgG (M5899; Sigma, St. Louis, MO, USA) was dispensed at a dose of 2.0 µg cm^−1^ at the position two cm away from the dipping point. For the test line, capture antibody (10-2698 from Fitzgerald or HM026 from EastCoast Bio) was dispensed at a dose of 0.5–2.0 µg cm^−1^ at the position 1.5 cm away from the dipping point. The membrane was then dried for 2 h at 37 °C. An absorption pad (Extra Thick Blot Paper, BIO-RAD, Hercules, CA, USA) was applied to the dried membranes, which were then cut to a width of four mm by an Autokun cutter (Hangzhou Autokun Technology, Hangzhou, China). Finally, test strips were sealed in aluminum packages with a desiccation pad and stored at 4 °C until use. Three nitrocellulose membrane types were tested, namely *CNPC-SS12*, 10 µm with wicking time of 140 ± 28 s/40 mm (MDI Technologies, Ambala Cantt, India), UniSart^®^ CN140 with wicking time of 95–155 s/40 mm, and UniSart^®^ CN 95 with wicking time of 65–115 s/ 40 mm (Sartorius, Goettingen, Germany).

### Assay procedure

Briefly, 10 µl of sample was mixed with 90 µl of running buffer (PBS 1×; 0.5% BSA; 1% Tween^®^-20; (Sigma, St. Louis, MO, USA) pH = 7.4) and one to five µl of biotinylated, mAb-coated magnetic nanoparticles in a well of a low-binding 96-well plate (Corning, Corning, NY, USA) before being flowed vertically onto a LFIA strip. After 25 min, if brown signals are observed at both test line and control line, a positive result could be concluded. If the signal is only observed at the control line, a signal amplification step is carried out by dipping the test strip into a well containing 30 µl of Streptavidin-PolyHRP80 conjugate at the concentration of 1–10 ng ml^−1^ (Fitzgerald, Acton, MA, USA). The excess of Streptavidin-PolyHRP80 conjugate was washed by dipping the test strip into a new well containing 50 µl of the running buffer, followed by absorbent pad removal and application of 200 µl of 1-Step™ Ultra TMB-Blotting Solution (Thermo Fisher Scientific, Waltham, MA, USA) on the test strip. The enzyme-substrate reaction was maintained for 10 min at room temperature before reading the results.

### Analytical sensitivity and cross-reactivity

Analytical sensitivity of the magneto-enzyme LFIA was determined using recombinant NS1 of four dengue serotypes (The Native Antigen Company, Kidlington, UK). These recombinant antigens were produced in mammalian HEK293 cells (purity greater than 95%). Serial dilutions of each NS1 serotype were spiked into negative sera (confirmed by both RT-qPCR and Dengue IgG/IgM 3.0 Combo rapid test as described) and were subjected to analysis using the same procedure as above.

The cross-reactivity of the developed assay was tested using recombinant NS1 from Zika virus at the concentration of 100 ng ml^−1^; an HBV-positive serum, an HCV-positive serum, and a Japanese encephalitis virus-positive serum. The analytical procedure was carried out the same as specified above.

### Data analysis

Data were analyzed according to Jacinto et al. (2018). For optimization experiments, test strips were captured by a Perfection V600 scanner (Epson, Suwa, Japan). Optical densities of test lines and control lines were digitalized to obtain signal values using ImageJ software (ver.1.47; HIH, Bethesda, MD, USA) (Schneider, Rasband & Eliceiri, 2012). The images were converted to 32-bit grayscale to acquire gray level intensities. A 40 × 140 pixels sq. region of interest was used to assess the signal intensities, which were then averaged by taking three or eight replications. GraphPad Prism 6.0 (GraphPad Software Inc., San Diego, CA, USA) was used to statistically analyze and graph the data. The LOD for each dengue serotype was determined as mean + 3SD of blank sample. Unpaired, two-tailed *t*-tests were performed to determine statistical significance. For testing clinical performance, results were read with the naked eye.

## Results

### Principle of the magneto-enzyme lateral flow immunoassay

The concept of the magneto-enzyme LFIA developed in this study is to use biotinylated, mAb-conjugated magnetic nanoparticles as the detection complex. Biotin molecules on this complex allow subsequent binding with Streptavidin-polyHRP conjugates, generating amplified signals when TMB substrate is added.

**Figure 1:**
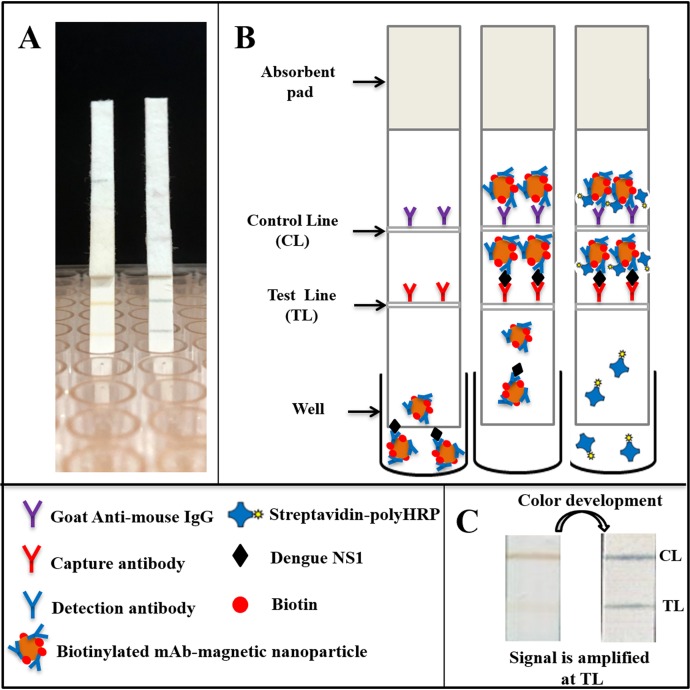
Schematic representation of the magneto-enzyme LFIA. (A) NS1-positive test strips without and with the signal amplification step. (B) The principle of the magneto-enzyme LFIA with associated legends. Briefly, the sample is diluted 10-fold in the running buffer and mixed with biotinylated, mAb-conjugated magnetic nanoparticles in a well of a 96-well plate. The mixture is then flowed onto a test strip, on which the capture antibody and anti-mouse IgG are immobilized at the test line and control line, respectively. The result could be read with the naked eye within 25 min. If no or faint test line is observed, a signal amplification step is performed by flowing Streptavidin-PolyHRP conjugates onto the test strip, followed by a washing step and color development by adding TMB substrate in order to confirm negative result. (C) Images of a typical test strip that requires the signal amplification step.

The assay consists of two main steps: (i) sample analysis on the lateral flow strip and (ii) signal amplification (Fig. 1). Specifically, sera of suspected dengue patients are diluted 10-fold in running buffer and mixed with biotinylated, mAb-coated magnetic nanoparticles in a well of a low-binding 96-well plate. The mixture was then allowed to flow onto the test strip by capillary force. When NS1-mAb-magnetic nanoparticle complexes reach the test line position, antigen-antibody sandwiches will be formed, consisting of capture antibodies, NS1, and the biotinylated magnetic nanoparticles. If dengue NS1 exists in high concentration in the specimens, test line signal could be observed directly (within 25 min). In case of low NS1 concentration (faint or no test line observed), a signal amplification step could be performed by flowing Streptavidin-polyHRP conjugates onto the test strip, washing off all the non-bound HRP conjugates and color development by TMB addition (total assay time of 90 min). Goat anti-mouse IgG, immobilized at the control line position, will always generate a signal of brown color, independent of the presence of dengue NS1 in the specimens.

### Optimization of the magneto-enzyme lateral flow immunoassay

The performance of magneto-enzyme LFIA strongly depends on (1) antibodies used, (2) magnetic nanoparticles and nitrocellulose membranes, (3) capture antibody application, (4) amounts of detection complexes. Therefore, the effects of these parameters on the test performance were determined.

**Figure 2:**
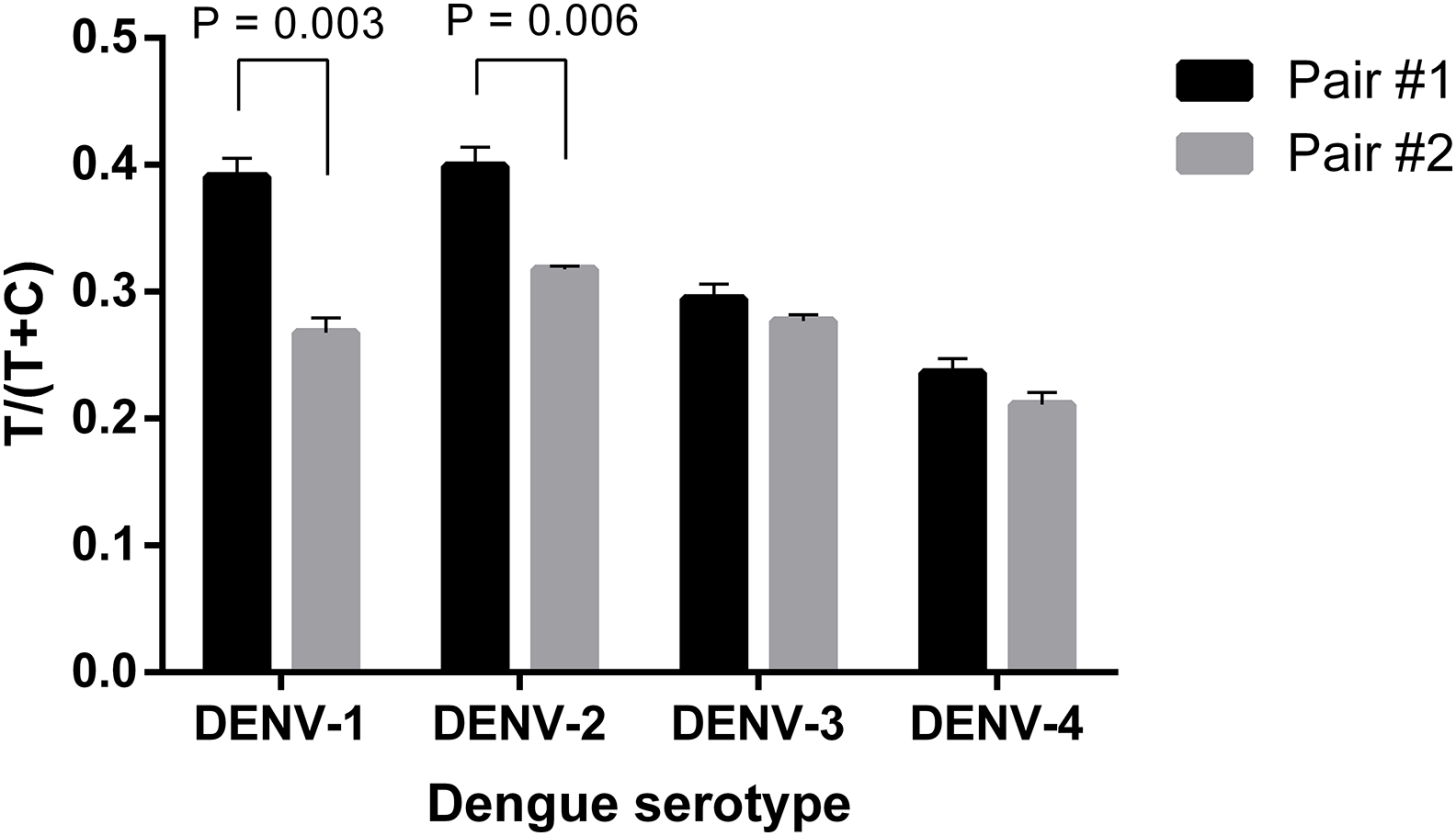
Selection of antibody pair for LFIA detecting dengue NS1. The performance of pair #1 (10-2698: capture antibody, 10-2699: detection antibody) and pair #2 (HM026: capture antibody, HM164: detection antibody) was evaluated for detection of 20 ng ml^−1^ NS1 of DENV-1, -2, -3, -4. Signal intensities were quantified and normalized by T/(T+C). T, test line signal; C, control line signal.

Firstly, two antibody pairs from Fitzgerald (10-2698: capture antibody and 10-2699: detection antibody; pair #1) and EastCoast Bio (HM026: capture antibody and HM164: detection antibody; pair #2) were tested. At the same concentration of 20 ng ml^−1^, all the four DENV serotypes were detected using the two antibody pairs. However, pair #1 produced higher signal intensities, especially when detecting DENV-1 and 2 (Fig. 2; Fig. S1). Therefore, 10-2698 and 10-2699 (Fitzgerald) were chosen to be immobilized at the test line position and conjugated to magnetic nanoparticles, respectively. Since DENV-4 generated the lowest signal intensities, further optimizations were carried out using DENV-4 NS1.

**Figure 3:**
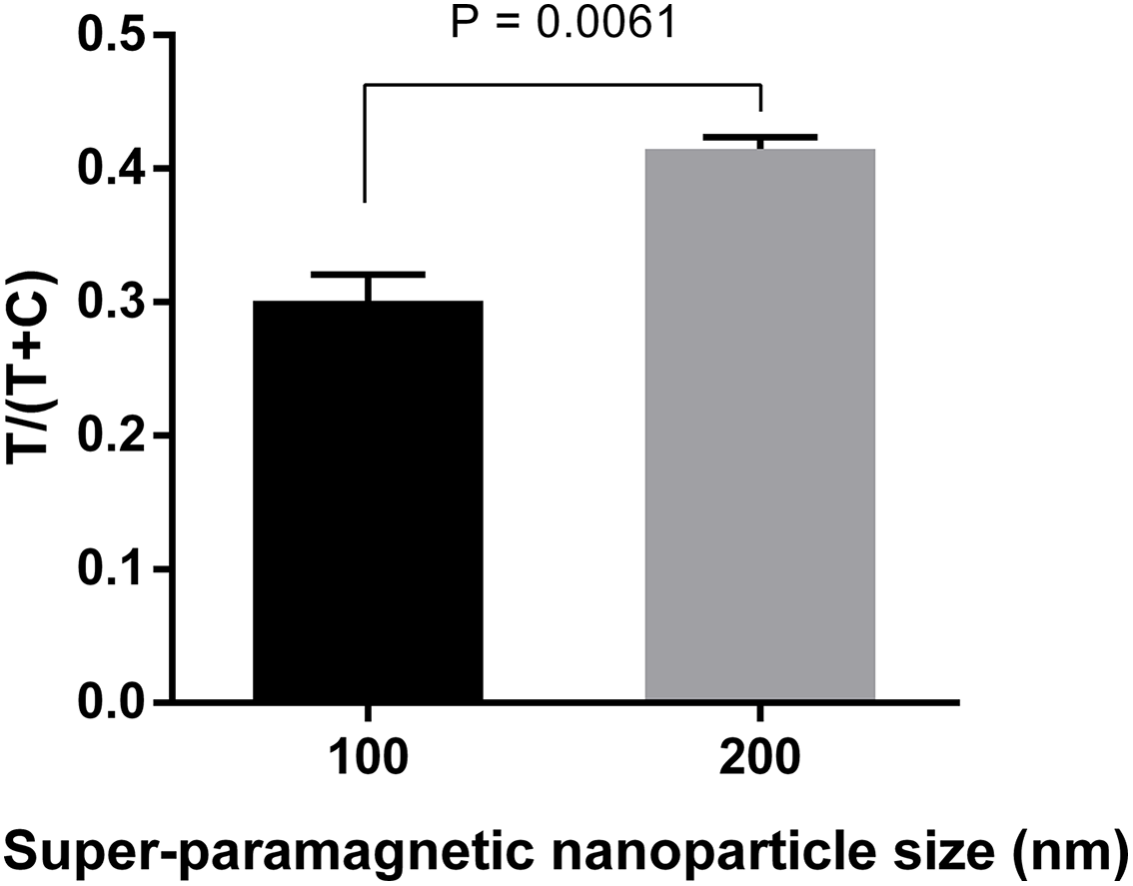
Optimization of super-paramagnetic nanoparticle size for LFIA detecting dengue NS1. Detection conjugates prepared from 100-, 200-, and 300 nm- Carboxyl-Adembeads were mixed with 50 ng ml^−1^ DENV-4 NS1 and flowed onto LFIA test strips. Nanoparticles of 300 nm were not able to migrate properly along the test strips, therefore, only signals generated by 100 and 200 nm super-paramagnetic nanoparticles were quantified. T, test line signal; C, control line signal.

Three different sizes (100, 200, and 300 nm) of Adembeads super-paramagnetic nanoparticle were tested. Immunocomplexes prepared from 300 nm Carboxyl-Adembeads were not able to migrate properly along the strip and caused aggregation at the dipping zone, which affected the readout (Fig. S2). Therefore, they were excluded from further analysis. Figure 3 clearly showed that super-paramagnetic nanoparticles with diameter of 200 nm displayed the best performance.

**Figure 4:**
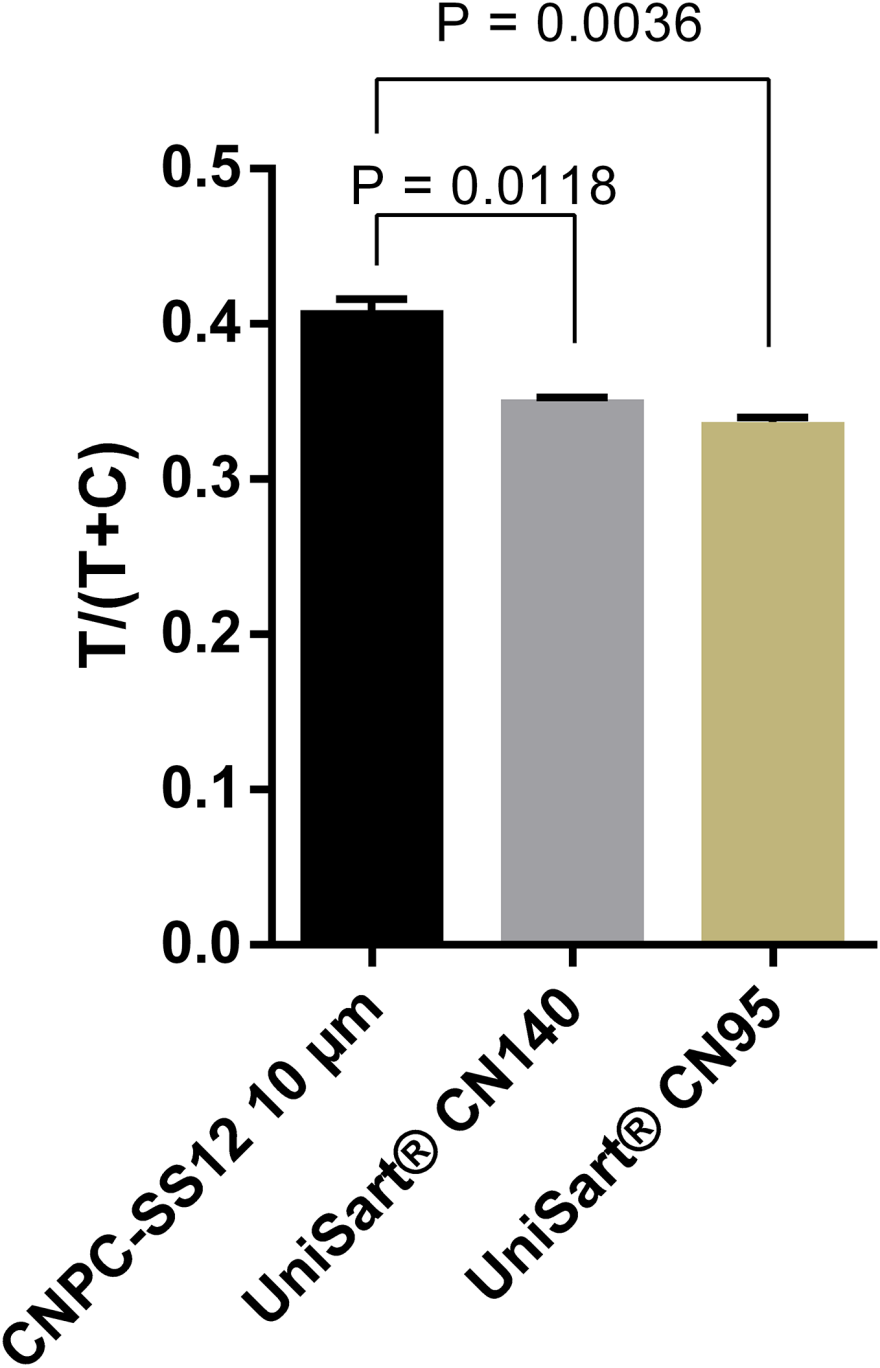
Selection of nitrocellulose membrane. Quantification of signal intensities of positive samples (50 ng ml^−1^ DENV-4 NS1) on three different nitrocellulose membrane types. T, test line signal; C, control line signal.

A total of three nitrocellulose membrane types were also evaluated. The highest signals were obtained with the membrane type CNPC-SS12, 10 µm (Fig. 4; Fig. S3). Therefore, “CNPC-SS12, 10 µm” from MDI technologies was chosen for subsequent experiments.

**Figure 5:**
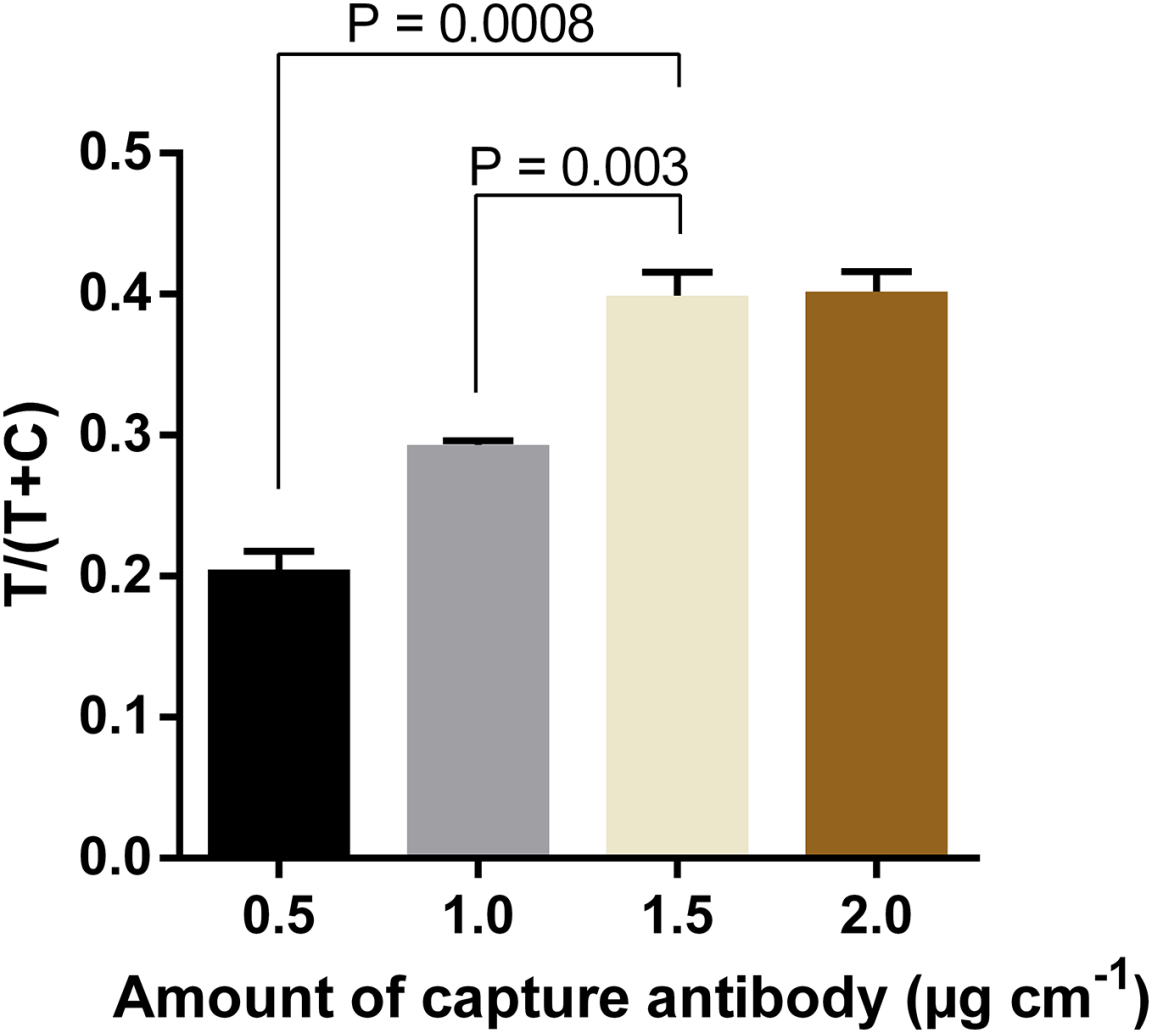
Optimization of amounts of capture antibody to be immobilized at the test line. Quantification of signal intensities of DENV-4 NS1 (50 ng ml^−1^) on LFIA strips when different amounts of anti-NS1 capture antibody were used. T, test line signal; C, control line signal.

In order to further increase the intensity of test line signals, the optimal amount of capture antibody to be immobilized at the test line was determined. Results showed that the intensity increased proportionally with the amount of antibody used, and reached the saturation when the capture antibody was immobilized at 1.5 µg cm^−1^ (Fig. 5; Fig. S4). The difference in signal intensity between immobilization of capture antibody at 1.5 µg cm^−1^ and that at 2.0 µg cm^−1^ was not statistically significant (*P* = 0.909).

**Figure 6:**
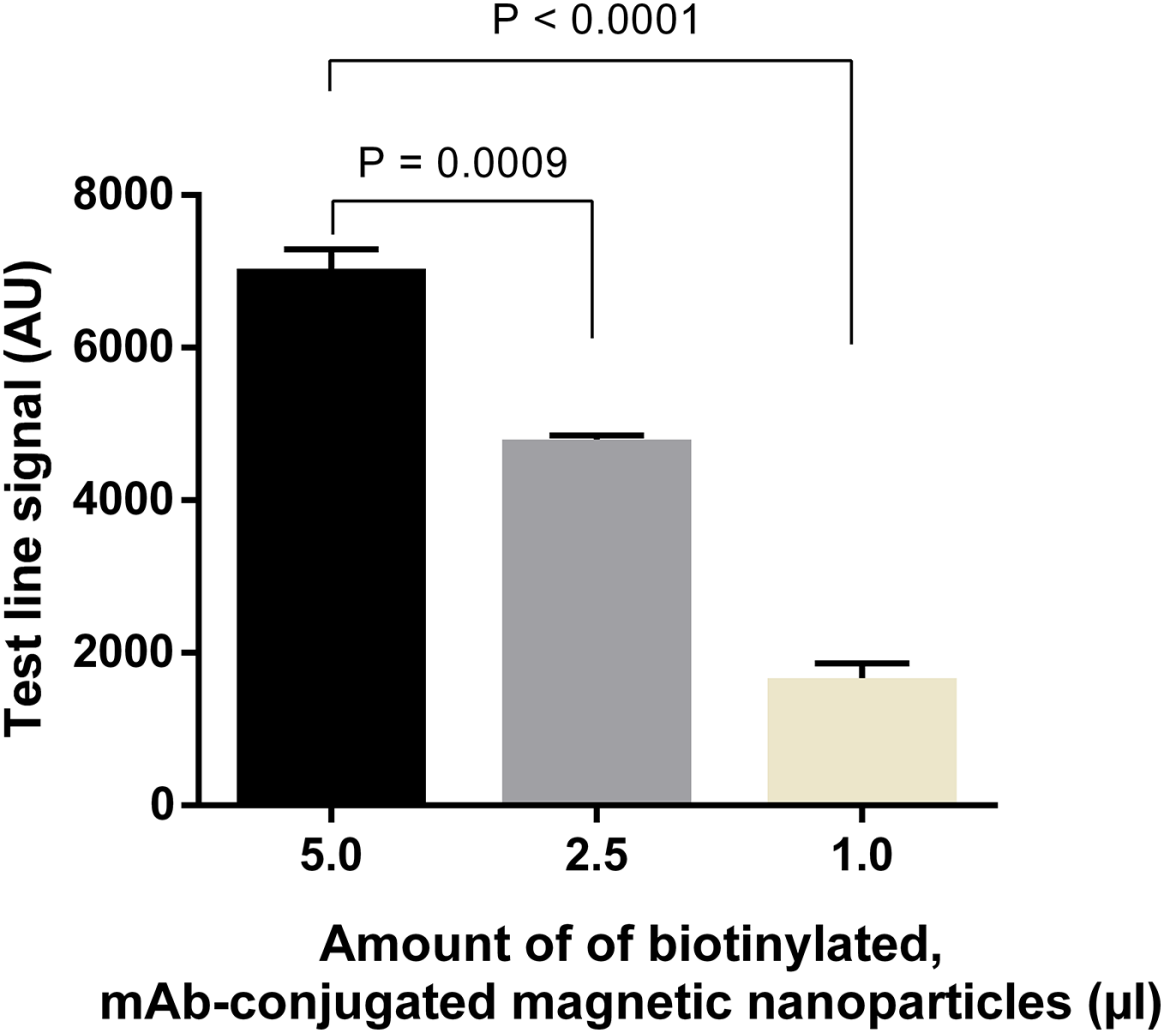
Optimization of amounts of biotinylated, mAb-connjugated magnetic nanoparticles. Different amounts of biotinylated, mAb-conjugated magnetic nanoparticles were mixed with 50 ng ml^−1^ DENV-4 NS1 before analysis on LFIA test strips. Signal intensities at test lines were then quantified for each experimental condition. AU, arbitrary unit.

**Figure 7:**
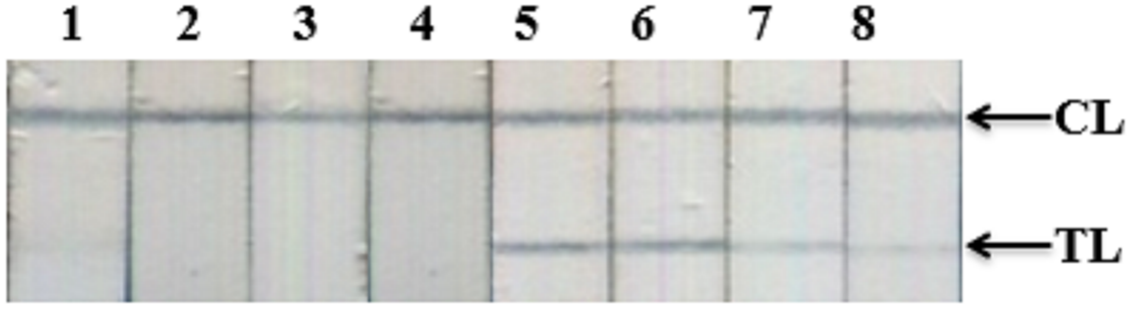
Optimization of amounts of Streptavidin-polyHRP conjugate for signal amplification. Detection of DENV-4 NS1 on representative LFIA strips when different amounts of Streptavidin-polyHRP were used; 1, 5, negative control and positive sample (5 ng ml^−1^) with 10 ng ml^−1^ of Streptavidin-polyHRP; 2, 6, negative control and positive sample with 5.0 ng ml^−1^ of Streptavidin-polyHRP; 3, 7, negative control and positive sample with 2.0 ng ml^−1^ of Streptavidin-polyHRP. 4, 8, negative control and positive sample with 1.0 ng ml^−1^ of Streptavidin-polyHRP.

Similarly, amounts of mAb-magnetic bead complexes and Streptavidin-polyHRP conjugate were optimized. Increasing the amount of mAb-magnetic bead complexes could enhance signal intensities at the test line (Fig. 6; Fig. S5). From the results presented, five µl of biotinylated mAb-magnetic bead complexes were used for detection of NS1 antigen in the next experiments. For the Streptavidin-polyHRP conjugate, various concentrations ranging from one to 10 ng ml^−1^ were tested. High streptavidin-polyHRP concentration could enhance signal intensities at the test line (Fig. 7; Fig. S6). However, false-positive results were observed at the highest concentration tested (10 ng ml^−1^). As a result, the optimal concentration of HRP conjugates was five ng ml^−1^.

### Analytical sensitivity

**Figure 8:**
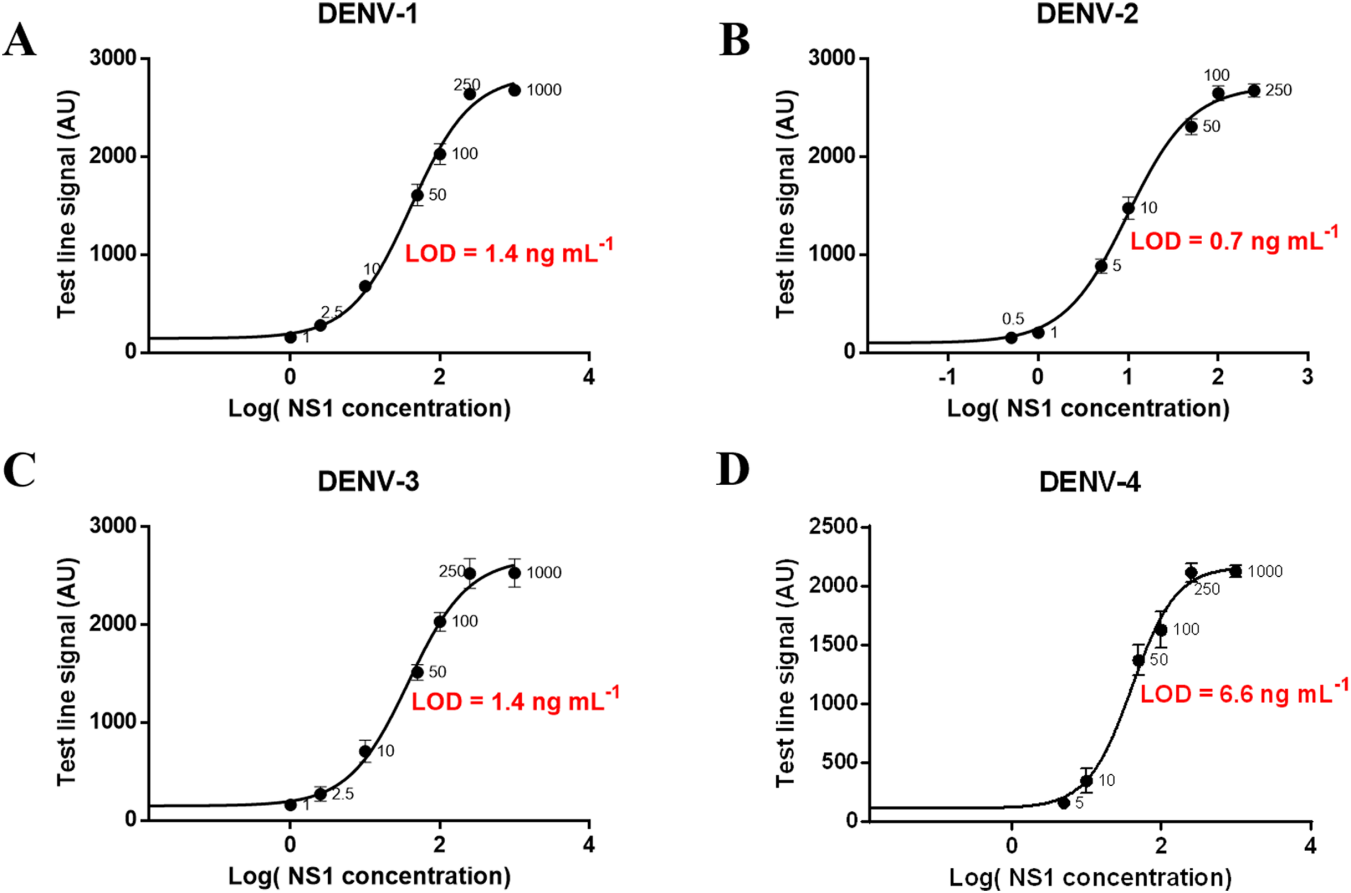
Calibration curves and LOD of the developed assay for detecting NS1 antigen of DENV-1, DENV-2, DENV-3, and DENV-4. Calibration curves and LOD of the developed assay for detecting NS1 antigen of DENV-1 (A), DENV-2 (B), DENV-3 (C), and DENV-4 (D). The error bars represent the standard deviation of eight independent experiments. The LOD was defined as mean + 3SD of blank sample.

Different concentrations of NS1 antigen were spiked into negative sera to determine the analytical sensitivity of the magneto-enzyme LFIA. The assay could detect directly (without the signal amplification step) NS1 at 1.0 ng ml^−1^ for DENV-2; 2.5 ng ml^−1^ for DENV-1 and DENV-3; and 10 ng ml^−1^ for DENV-4 when observed with the naked eye (Figs. S7 and S8). With the additional signal amplification step, sensitivities were increased by 10-fold for all four serotypes: 0.1 ng ml^−1^ for DENV-2; 0.25 ng ml^−1^ for DENV-1 and DENV-3; and one ng ml^−1^ for DENV-4 (Fig. S9). A calibration curve of log(NS1 concentration) vs. test line signal was also plotted for each dengue serotype and the theoretical LOD was defined as mean of the blank + 3SD. Figure 8 showed that the LODs of our assay (without the signal amplification step) for NS1 from DENV-1, DENV-2, DENV-3, and DENV-4 were approximately 1.4, 0.7, 1.4, and 6.6 ng ml^−1^ respectively. Of note, LOD of the gold nanoparticle-based LFIA using the same antibody pair was 7.9 ng ml^−1^ for DENV-2 NS1 (Fig. S10). These results revealed that the use of 200 nm Carboxyl-Adembeads enhanced the analytical sensitivity of LFIA. To our knowledge, the assay developed in this research is the most sensitive NS1 test to date. Before this study, dengue NS1 (serotype 1) could be identified at the lowest concentration of 4.9 ng ml^−1^ (Kumar et al., 2018).

### Cross-reactivity tests

**Figure 9:**
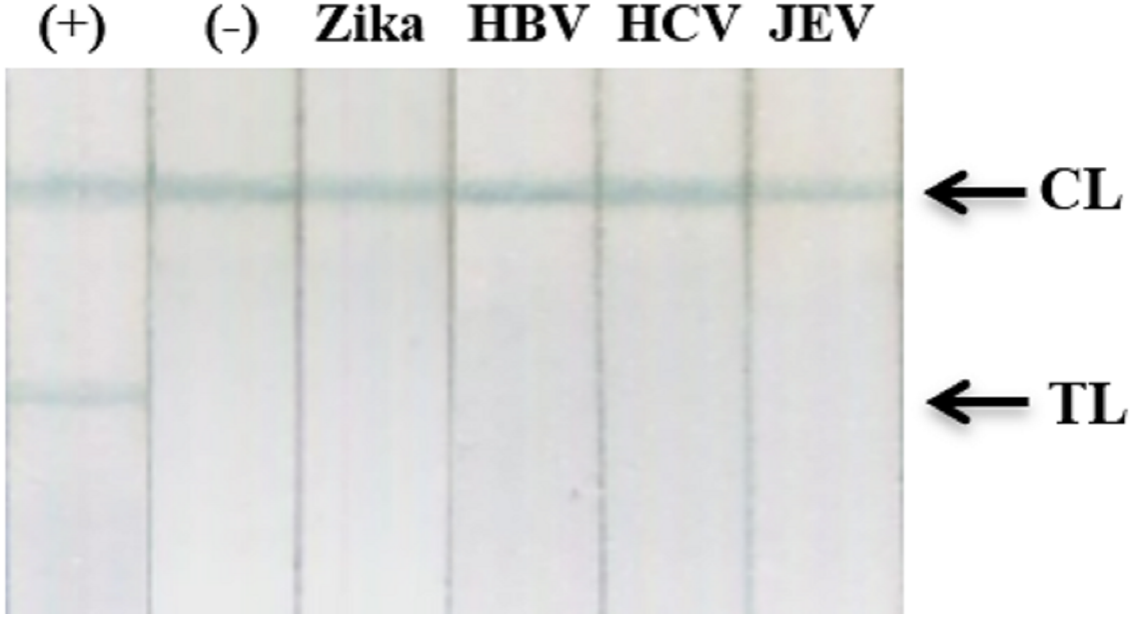
Cross-reactivity test against recombinant Zika NS1, HBV, HCV, and JEV clinical samples. (−), negative control; (+) DENV-2 NS1 (1 ng ml^−1^); Zika, recombinant NS1 of Zika virus (100 ng ml^−1^); HBV, HCV, clinical sera of Hepatitis B virus (viral load = 2.2 × 10^5^ IU/ml), and Hepatitis C virus (viral load = 4.3 × 10^4^ IU/ml); JEV, clinical serum positive with Japanese encephalitis virus IgM antibodies; TL, test line; CL, control line.

The specificity of our assay was assessed using recombinant NS1 from Zika virus and clinical samples with high viral loads of HBV, HCV, or Japanese encephalitis virus. Results showed an absence of cross-reactivity, even with NS1 from Zika virus (Fig. 9; Fig. S11).

### Clinical performance

Among 120 clinical sera, six samples were found positive for both RT-qPCR and Dengue IgG/IgM 3.0 Combo rapid test, 64 samples were only found positive for RT-qPCR, and 50 samples were found negative by both tests (Table S1). Typing of positive samples by nested RT-PCR (Lanciotti et al., 1992) showed that 11 (15.7%) were positive with DENV-1, 55 (78.6%) were positive with DENV-2, and 4 (5.7%) were positive with DENV-4 (Fig. S12). This panel was then used to compare the analytical performance of the developed magneto-enzyme LFIA to that of Dengue Ag rapid test CE kit (CTK Biotech, Inc., Poway, CA, USA). The commercial assay detected only 66 out of 70 positive samples. By contrast, the magneto-enzyme LFIA developed in this study was able to detect all 70 positive sera from dengue-infected patients (Fig. S13). Of note, signal amplification by polyHRP-TMB reaction allowed the detection of four positive specimens that were found negative by the commercial assay (Fig. S13B). Moreover, these four samples were negative for IgM and IgG by Dengue IgG/IgM 3.0 Combo rapid test (CTK Biotech, Inc., Poway, CA, USA). Therefore, our assay is likely to be able to diagnose primary dengue infection at the very first days of the disease. Besides, no false positive results were obtained by both the commercial assay and the magneto-enzyme LFIA (Tables 1 and  2). To summarize, our magneto-enzyme assay showed higher sensitivity (100% vs. 94%) and accuracy (100% vs. 97%) than Dengue Ag rapid test CE (CTK Biotech, Poway, CA, USA); while the specificities of the two assays were equivalent (100%) (Table 3). These results also indicated that the developed assay could accurately detect DENV-1, -2, -4 NS1 in clinical samples.

**Table 1:** Results of 120 clinical sera tested with Dengue Ag rapid test CE compared to RT-qPCR combined with Dengue IgG/IgM 3.0 Combo rapid test.

		RT-qPCR and Dengue IgG/IgM 3.0 Combo rapid test (*n* = 120)	
		Positive (*n* = 70)	Negative (*n* = 50)
Dengue Ag rapid test CE	Positive	66	0
	Negative	4	50

**Table 2:** Results of 120 clinical sera tested with magneto-enzyme LFIA compared to RT-qPCR combined with Dengue IgG/IgM 3.0 Combo rapid test.

		RT-qPCR and Dengue IgG/IgM 3.0 Combo rapid test (*n* = 120)	
		Positive (*n* = 70)	Negative (*n* = 50)
Magneto-enzyme LFIA	Positive	70	0
	Negative	0	50

**Table 3:** Comparison of performance parameters of the magneto-enzyme LFIA with those of Dengue Ag rapid test CE (CTK Biotech, Poway, CA, USA).

Assays	Sensitivity (%)	Specificity (%)	Accuracy (%)
Dengue Ag rapid test CE (CTK Biotech, Poway, CA, USA)	94	100	97
Magneto-enzyme LFIA	100	100	100

## Discussion

Dengue NS1 is a non-structural glycoprotein, associates as a dimer to cell membranes and is secreted into the blood as a hexameric lipoprotein particle (Muller & Young, 2013). Dengue NS1 could be detected by ELISA in the bloodstream of the infected patients from the first day of symptom onset up to day 9. Its concentration could reach up to 50 µg ml^−1^ in the sera of some DENV-infected patients (Alcon et al., 2002). Therefore, NS1 plays an essential role in early diagnosis of DENV infections (Bessoff et al., 2008; Blacksell et al., 2011; Datta & Wattal, 2010; Dussart et al., 2006; Kassim et al., 2011; Tricou et al., 2011).

Initially, similar to the pioneering research of Cho & Irudayaraj (2013), the magneto-enzyme LFIA in this study consisted of three steps, namely immunomagnetic separation (IMS) with 1 h of incubation; LFIA; and signal amplification. Although this format allowed the detection of pure NS1 at the concentration as low as 0.02 ng ml^−1^ (data not shown), it caused bead aggregation at the dipping zone when tested with clinical sera. Consequently, the IMS step was removed from the procedure and samples were directly subjected to analysis on LFIA. Aggregation of super-paramagnetic nanoparticles on LFIA test strips was also observed by Wang et al. (2015). However, bead aggregation was not reported in the study of Cho & Irudayaraj (2013). This discrepancy could be due to the difference in sample matrices (milk samples vs. serum samples) and the size of the nanoparticle used (200 nm in our study vs. 30 nm in Cho & Irudayaraj (2013)). Although the use of SHP-30 carboxyl iron oxide nanoparticles could avoid bead aggregation, magnetic separation time would be too long for clinical application (up to 8 h) (Anonymous, 2017).

Several commercial immunochromatographic tests for the rapid detection of DENV NS1 are available, such as Dengue NS1 Ag STRIP™ (Bio-Rad, Hercules, CA, USA), SD BIOLINE™ Dengue Duo (Abbott, Santa Clara, USA; former Alere Inc, Waltham, USA), and Dengue Ag rapid test CE (CTK Biotech, Poway, CA, USA). The advantages of these test comparing to ELISA and RT-PCR assays are simplicity and rapidity. Nevertheless, limited sensitivities of these commercial rapid tests were found in several cases (Osorio et al., 2010). In the present study, we have developed a signal amplification step to improve the sensitivity of LFIA for NS1 detection. This new assay allows highly-sensitive detection of NS1 with a detection limit as low as 0.1 ng ml^−1^ for DENV-2, a nearly 50-fold improvement over the value recently reported by Kumar et al. (2018). Of note, DENV-2 is the most prevalent serotype detected in our study and commonly associated with dengue hemorrhagic fever or severe dengue (Fried et al., 2010; Vicente et al., 2016). Therefore, the developed assay could represent an attractive diagnostic method to lower the risk of serious complications caused by this serotype.

The high analytical sensitivity of our assay is mainly due to the use of biotinylated detection complex and Streptavidin-polyHRP conjugates. This signal amplification system allowed a 10-fold increase in the sensitivity of our assay. Although this step prolongs the assay time to approximately one and a half hours, which is significantly longer in comparison to other commercial LFIAs, our magneto-enzyme LFIA was able to detect four false negative results by the commercial CTK Dengue Ag rapid test CE kit. As mentioned above, these four samples were negative for both anti-dengue IgG and anti-dengue IgM. Therefore, our assay could allow the detection of primary dengue infections, even in the very early stage of the disease. Of note, most positive samples (66/70) were detected directly by our assay without the need of the signal amplification step, resulting in readouts within 25 min.

Another critical factor that influences the sensitivity of our assay is the diameter of magnetic nanoparticles. If the particles could reach the detection zone, a larger bead size generates intense brown signals. Moreover, it allows more biotinylation sites, leading to improved signals after the amplification step by Streptavidin-polyHRP. On the other hand, a smaller size resulted in a better migrating capability along the test strip. In our study, Carboxyl-Adembeads with a diameter of 200 nm were found to be optimal, producing high signal intensities while retaining the ability to migrate properly on the membrane. In comparison to the standard gold nanoparticle-based LFIA, using 200 nm Carboxyl-Adembeads allowed an 11-fold improvement in sensitivity for the detection of DENV-2 NS1. It is notable that Wang et al. (2013) have successfully used the 300 nm Carboxyl-Adembeads for their super-paramagnetic lateral-flow immunoassay to detect *Bacillus anthracis*. This discordance might be due to the difference in the nitrocellulose membranes used between studies.

In other magnetic LFIA platforms, signals at test lines and control lines could be quantified using a Magnetic Reader, which lower the LOD of the assay. However, this equipment is only available from few manufacturers (FoodChek™ MICT; MagnaBioSciences, San Diego, CA, USA; Magnia^Â^^®^ Reader; Magnasense, Vantaa, Finland) at a very costly price (more than 20,000 USD for FoodChek™ MICT). Therefore, these specialized magnetic readers are not applicable in clinical settings in developing countries. In order to eliminate the need for this specialized equipment, we have used a signal amplification strategy as described above. For most dengue-positive samples, which contain NS1 in high concentrations, this step is not required, and positive results could be obtained by our assay within 25 min. For patients infected with dengue at the early phase, NS1 may be present in low concentration, causing false negative results by conventional LFIAs. With the additional signal amplification step, the developed assay could detect a very low amount of NS1, thereby, preventing the false negative problem. Consequently, the magneto-enzyme LFIA developed in this study is particularly useful for the detection of dengue at the early stage of the disease, facilitating proper management of patients.

## Conclusions

In conclusion, we have successfully developed a magneto-enzyme LFIA that achieved a high sensitivity for the detection of dengue NS1. The LOD of our assay could reach as low as 0.1 ng ml^−1^ (for DENV-2), which is approximately 50 times lower than the best values previously reported. The new assay prospectively allows rapid and highly accurate diagnosis of dengue infections at point-of-care, which is imperative in management of patients and epidemic control.

## Supplemental Information

10.7717/peerj.7779/supp-1 Supplemental Information 1 Supplementary figures.

10.7717/peerj.7779/supp-2 Supplemental Information 2 Raw digital readouts for analytical sensitivity experiments (gold nanoparticle).

10.7717/peerj.7779/supp-3 Supplemental Information 3 Raw digital readouts for analytical sensitivity experiments.

10.7717/peerj.7779/supp-4 Supplemental Information 4 Raw digital readouts for optimization experiments.

10.7717/peerj.7779/supp-5 Supplemental Information 5 Testing results of dengue infection by RT-qPCR and Dengue IgG/IgM 3.0 Combo rapid test.

10.7717/peerj.7779/supp-6 Supplemental Information 6 Additional experiment results.

## Additional Information and Declarations

### Data Availability

The following information was supplied regarding data availability: The raw data are available in the Supplemental Files.
