# Supplementary material for: Development of a highly sensitive magneto-enzyme lateral flow immunoassay for dengue NS1 detection
Source: PeerJ. 2019 Sep 25;7:e7779. doi: 10.7717/peerj.7779 (PMC6765353; doi:10.7717/peerj.7779)
Supplement: Supplemental Information 1 [file peerj-07-7779-s001.pdf]

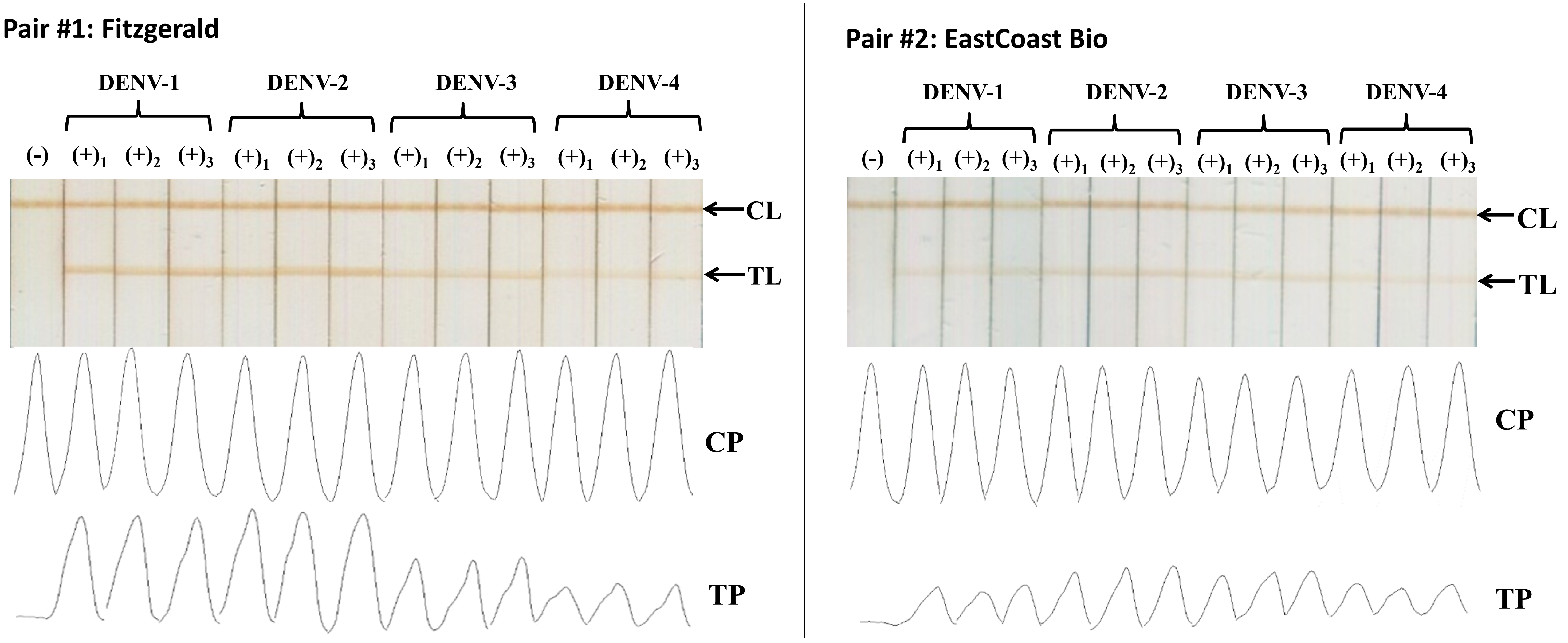

Figure S1: Selection of antibody pair for LFIA detecting dengue NS1

The performance of pair #1 (10-2698: capture antibody, 10-2699: detection antibody) and pair #2 (HM026: capture antibody, HM164: detection antibody) was evaluated for detection of 20 ng ml^{-1} NS1 of DENV-1, -2, -3, -4. The experiments were performed in triplicate. (-), negative control; (+), running buffer spiked with 20 ng ml^{-1} NS1; CL, control line; TL, test line. CP, ImageJ plots of control line of corresponding strips. TP, ImageJ plots of test line of corresponding strips.

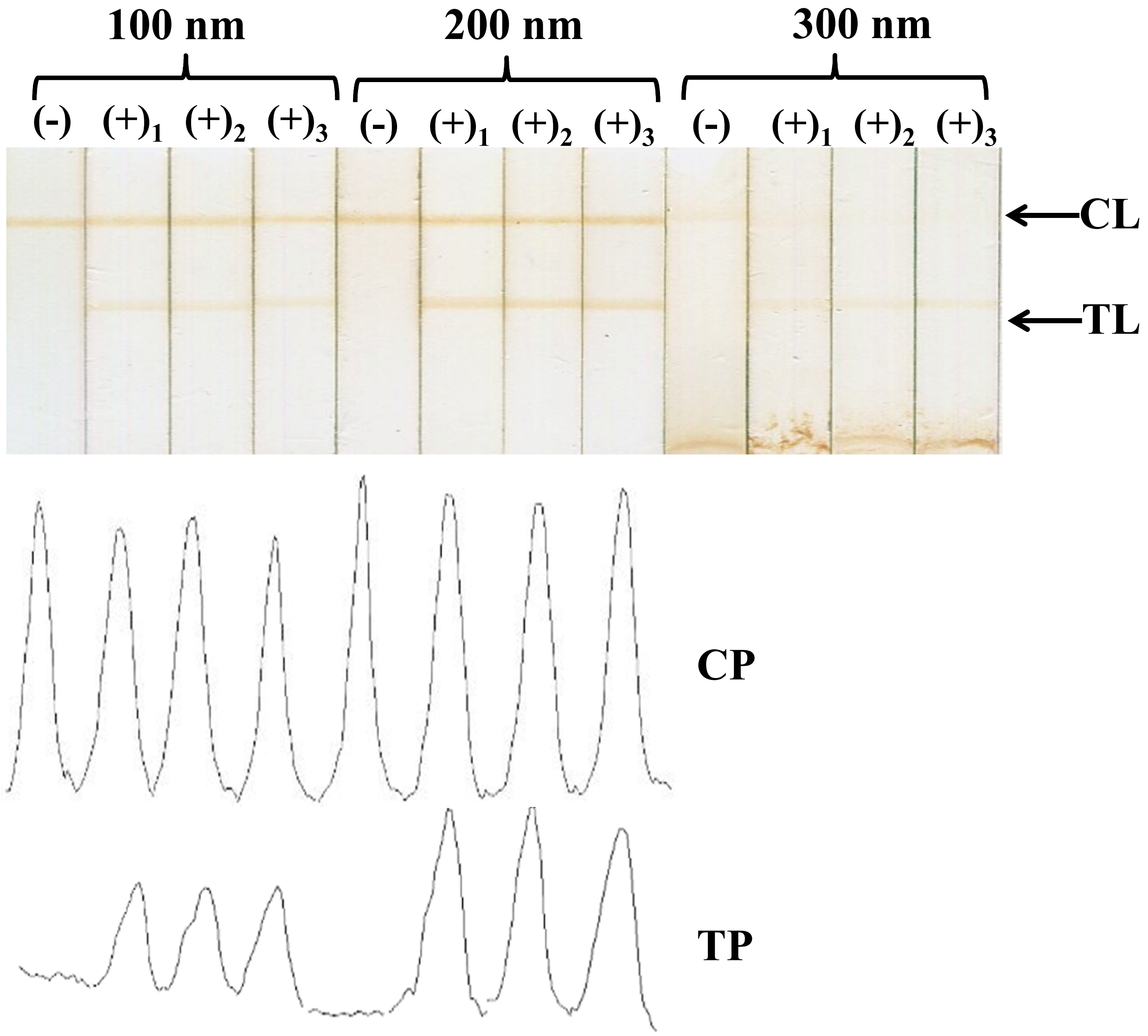

Figure S2: Optimization of super-paramagnetic nanoparticle size for LFIA detecting dengue NS1.
Negative controls and DENV-4 NS1 (50 ng ml^{-1}) were analyzed on LFIA strips using detection conjugates prepared from 100 nm-, 200 nm- and 300 nm- Carboxyl-Adembeads. (-), negative control; (+), running buffer spiked with 50 ng ml^{-1} DENV-4 NS1; CL, control line; TL, test line. CP, ImageJ plots of control line of corresponding strips. TP, ImageJ plots of test line of corresponding strips.

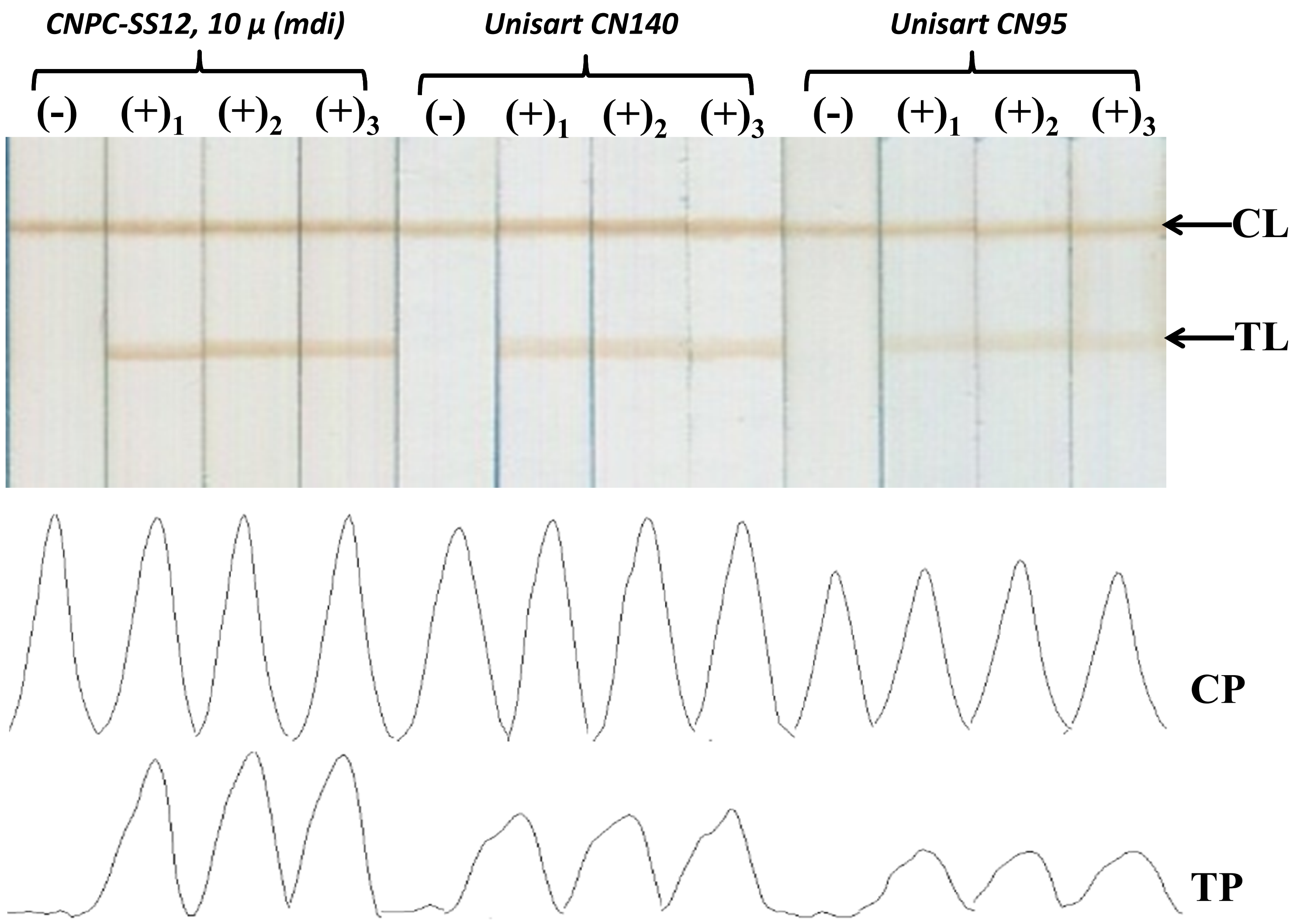

Figure S3: Selection of nitrocellulose membrane.
Negative controls and positive samples (50 ng ml^{-1} DENV-4 NS1) were analyzed on three different nitrocellulose membrane types. The experiment was performed in triplicate. (-), negative control; (+), running buffer spiked with 50 ng ml^{-1} DENV-4 NS1; CL, control line; TL, test line. CP, ImageJ plots of control line of corresponding strips. TP, ImageJ plots of test line of corresponding strips.

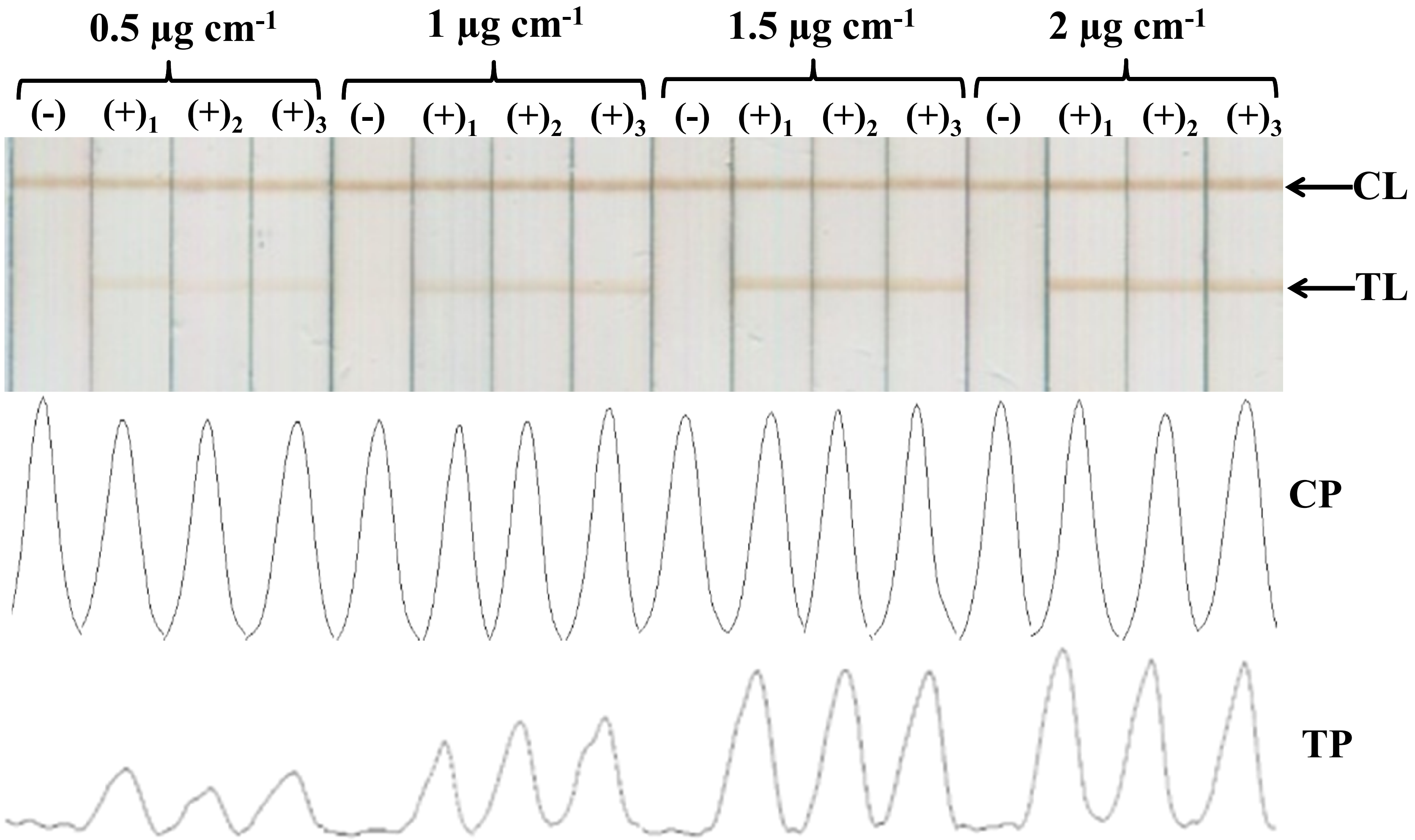

Figure S4: Optimization of amounts of capture antibody to be immobilized at the test line.
Negative controls and positive samples (50 ng ml^{-1} DENV-4 NS1) were analyzed on LFIA strips with different amounts of capture antibody. The experiment was performed in triplicate. (-), negative control; (+), running buffer spiked with 50 ng ml^{-1} DENV-4 NS1; CL, control line; TL, test line. CP, ImageJ plots of control line of corresponding strips. TP, ImageJ plots of test line of corresponding strips.

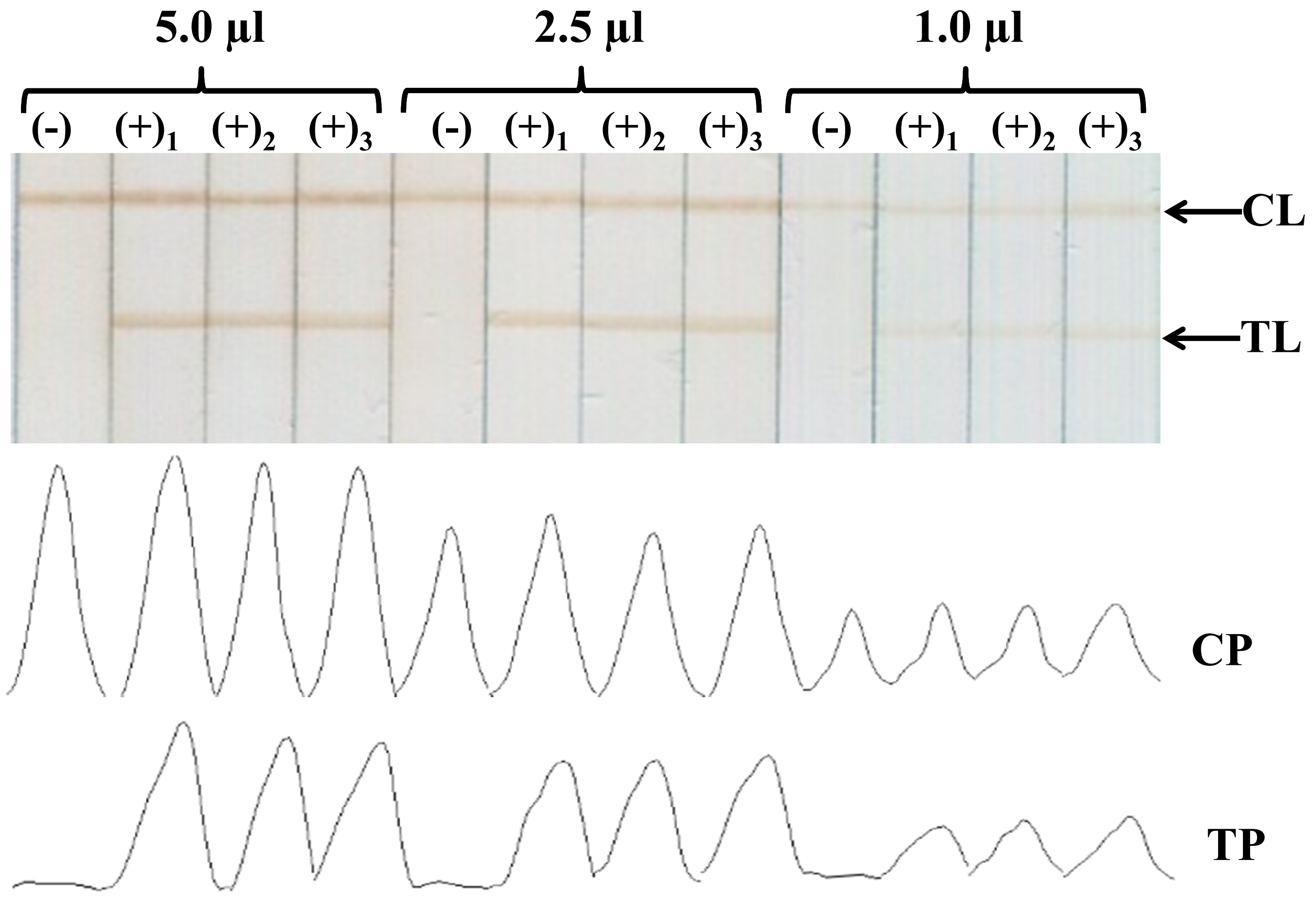

Figure S5: Optimization of amounts of biotinylated, mAb-conjugated magnetic nanoparticles.
Negative controls and positive samples (50 ng ml^{-1} DENV-4 NS1) were mixed with different amounts biotinylated, mAb-conjugated magnetic nanoparticles (5.0, 2.5, or 1.0 µl) and analyzed on LFIA strips. The experiment was performed in triplicate. (-), negative control; (+), running buffer spiked with 50 ng ml^{-1} DENV-4 NS1; CL, control line; TL, test line. CP, ImageJ plots of control line of corresponding strips. TP, ImageJ plots of test line of corresponding strips.

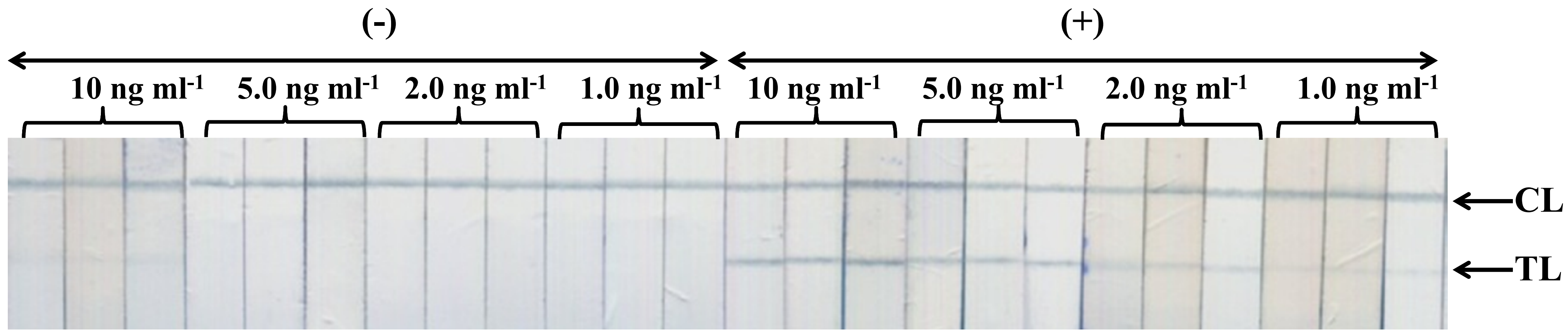

Figure S6: Optimization of amounts of Streptavidin-polyHRP conjugate for signal amplification
Negative controls and positive samples (5 ng ml^{-1} DENV-4 NS1) were analyzed on LFIAs and signals were amplified with 10 ng ml^{-1}, 5.0 ng ml^{-1}, 2.0 ng ml^{-1} or 1 ng ml^{-1} of Streptavidin-polyHRP. The experiment was performed in triplicate. (-), negative control; (+), running buffer spiked with 5 ng ml^{-1} DENV-4 NS1; CL, control line; TL, test line.

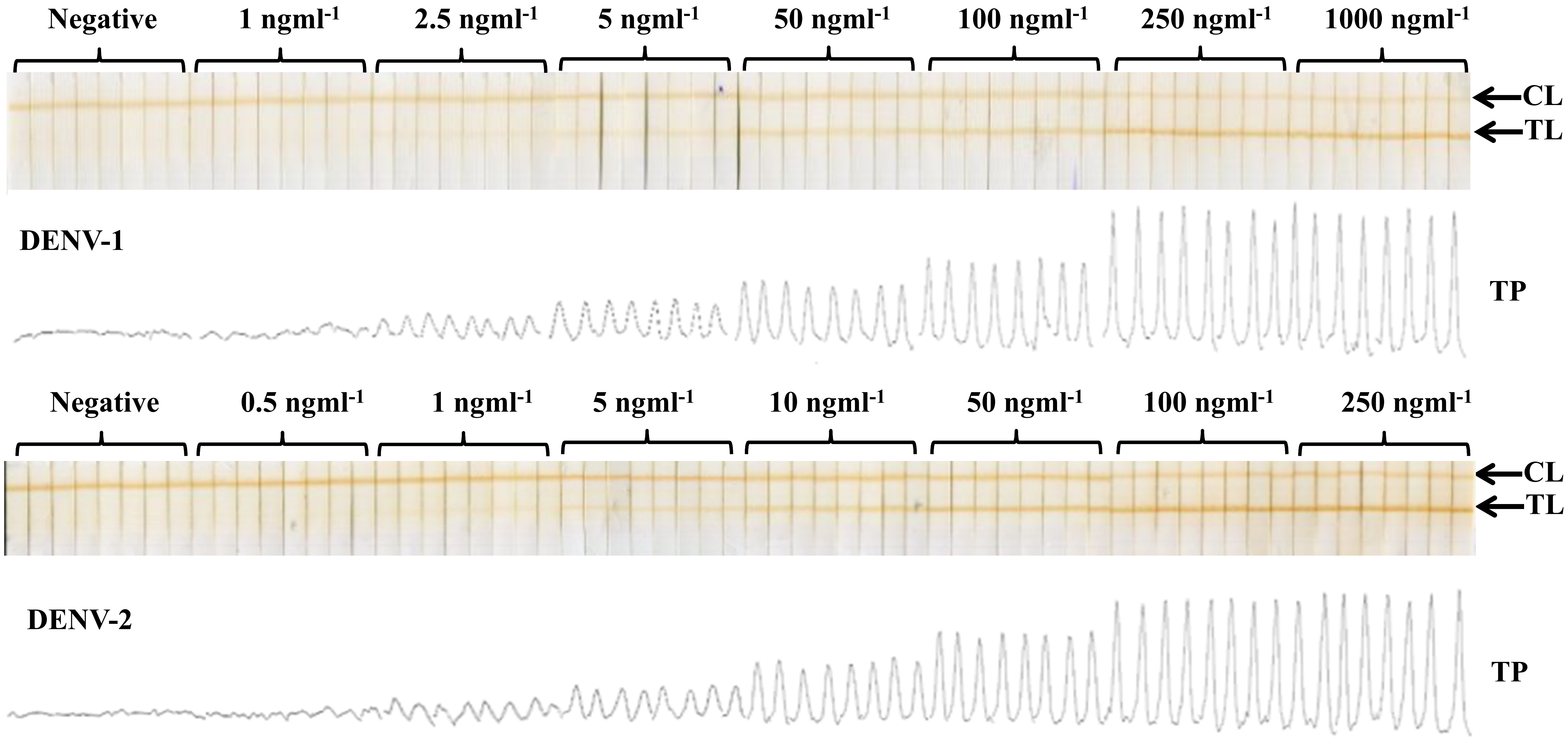

Figure S7: Analytical sensitivity of the magneto LFIA (without the signal amplification step) for detection of NS1 of DENV-1 and DENV-2.

The experiments were performed in 8 replicates. CL, control line; TL, test line. TP, ImageJ plots of test line of corresponding strips

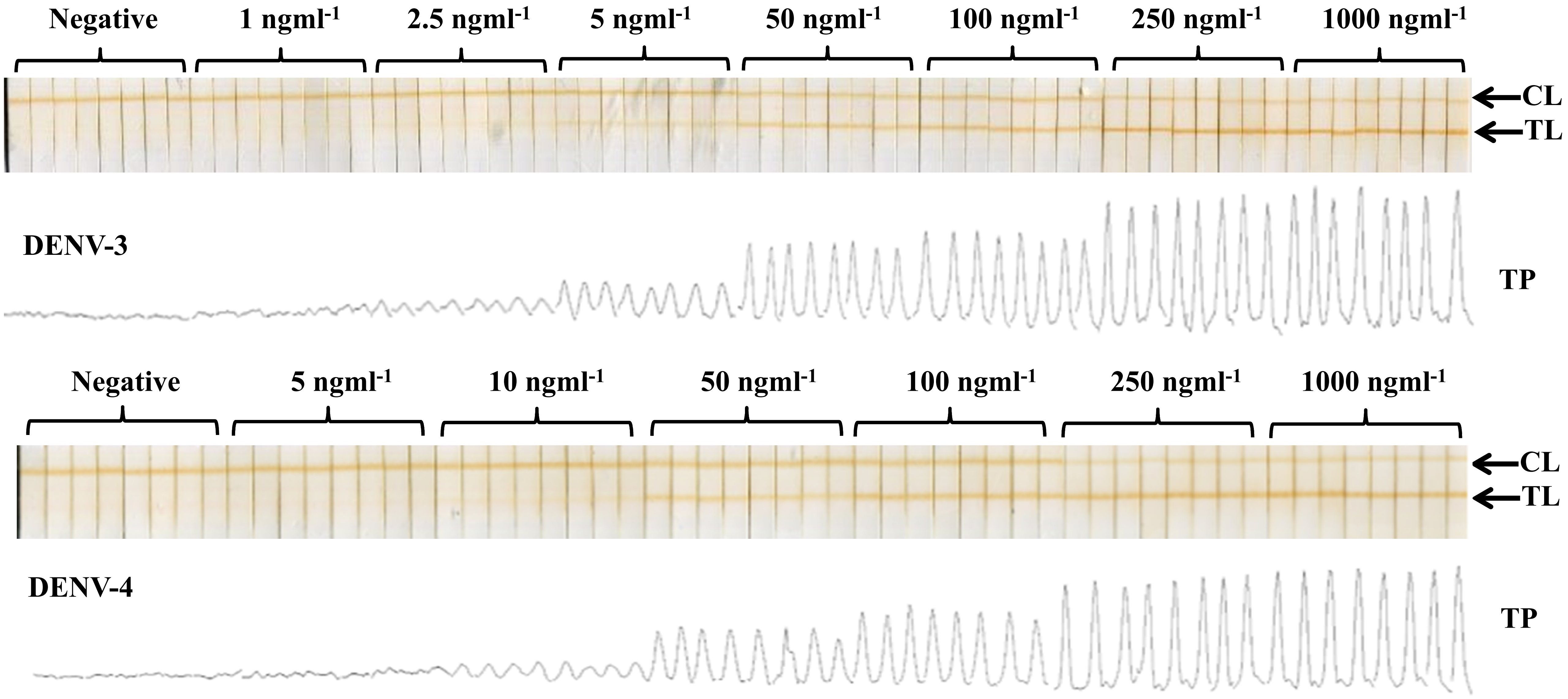

Figure S8: Analytical sensitivity of the magneto LFIA (without the signal amplification step) for detection of NS1 of DENV-3 and DENV-4.

The experiments were performed in 8 replicates. CL, control line; TL, test line. TP, ImageJ plots of test line of corresponding strips

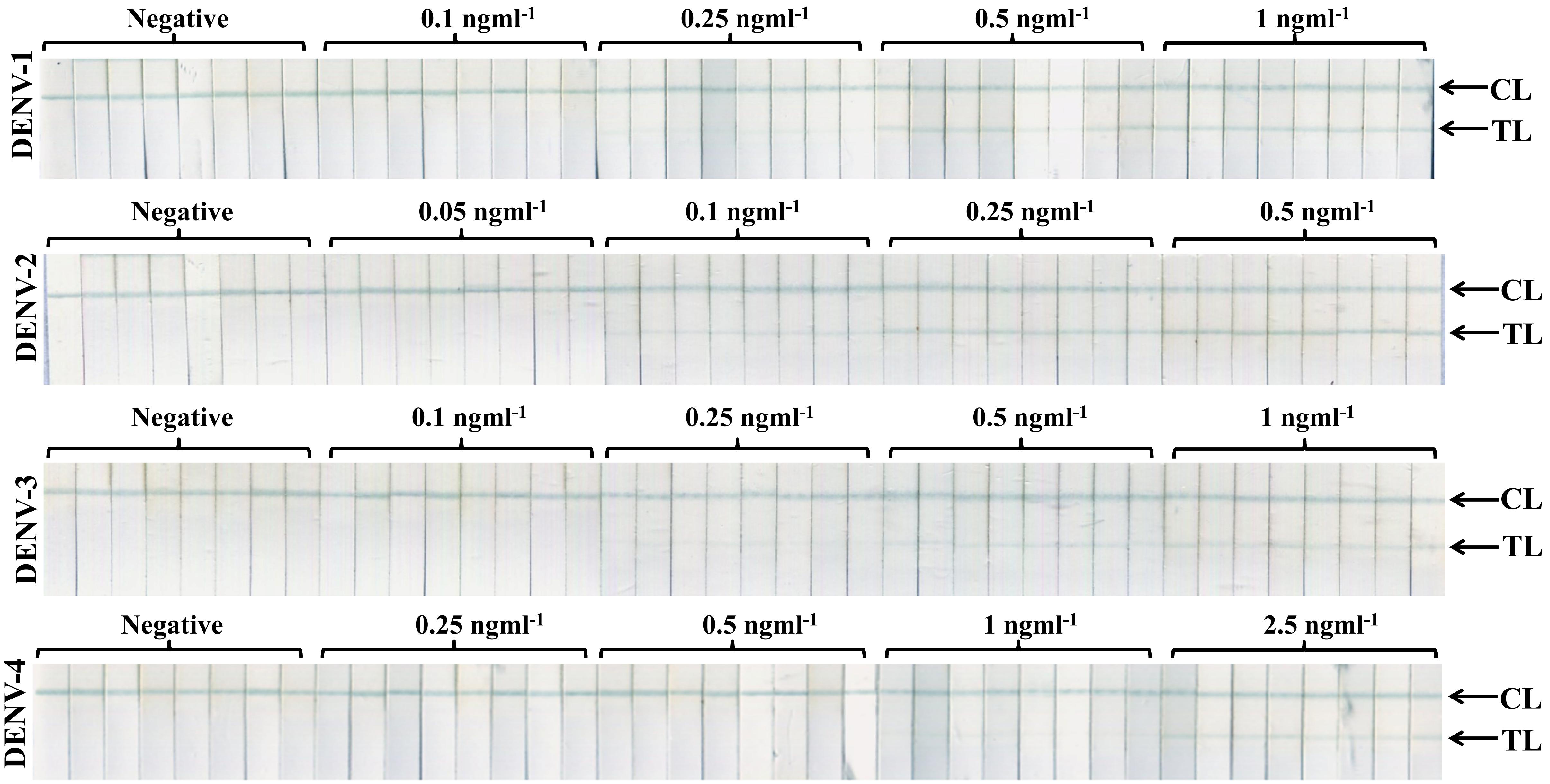

Figure S9: Analytical sensitivity of the magneto-enzyme LFIA (with the signal amplification step) for the detection of NS1 of DENV-1, -2, -3, -4.

The experiments were performed in 8 replicates. CL, control line; TL, test line.

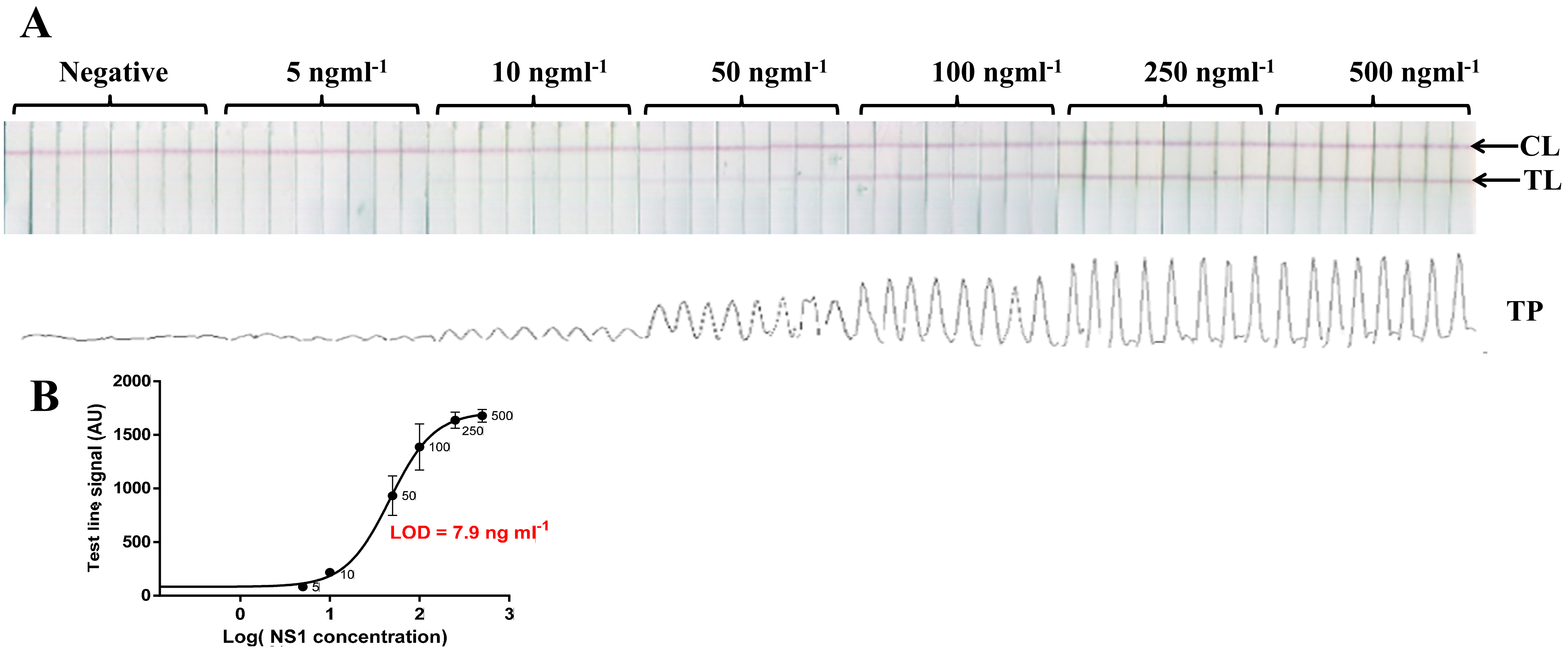

Figure S10: Analytical sensitivity of the standard 40 nm gold nanoparticle-based LFIA for detection of DENV-2 NS1. (A), images of test strips with gold nanoparticle conjugates. CL, control line; TL, test line. TP, ImageJ plots of test line of the corresponding strips. (B), calibration curve and LOD of the assay. The error bars represent the standard deviation of eight independent experiments. The LOD was defined as mean + 3SD of blank sample.

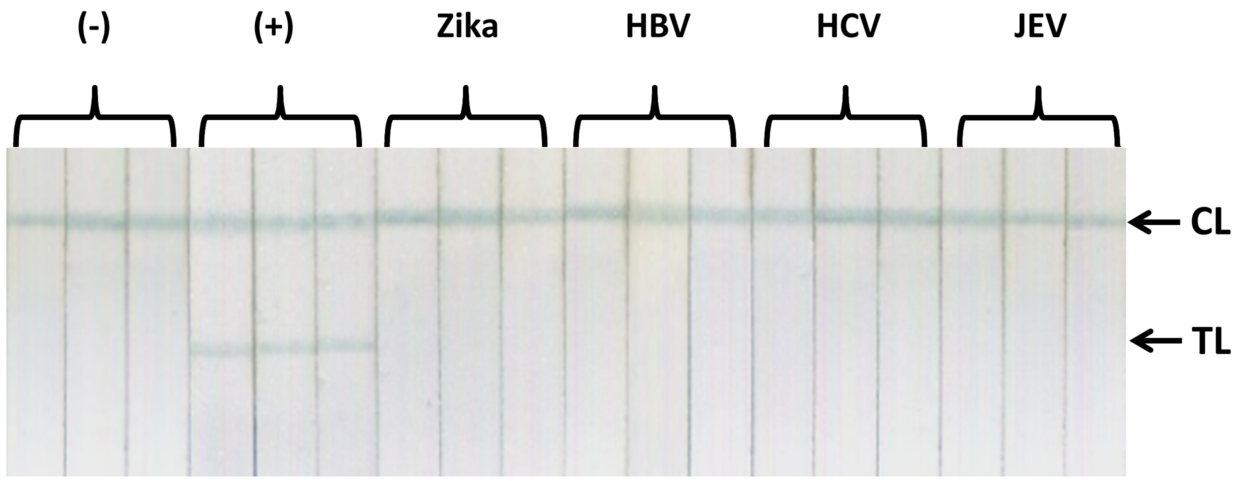

Figure S11: Cross-reactivity test against recombinant Zika NS1, HBV, HCV and JEV clinical samples
(-), negative control; (+) DENV-2 NS1 (1 ng ml^{-1}); Zika, recombinant NS1 of Zika virus (100 ng ml^{-1}); HBV, HCV, clinical sera of Hepatitis B virus (viral load = 2.2×10^5 IU ml^{-1}), and Hepatitis C virus (viral load = 4.3×10^4 IU ml^{-1}); JEV, clinical serum positive with Japanese encephalitis virus IgM antibodies; TL, test line; CL, control line.

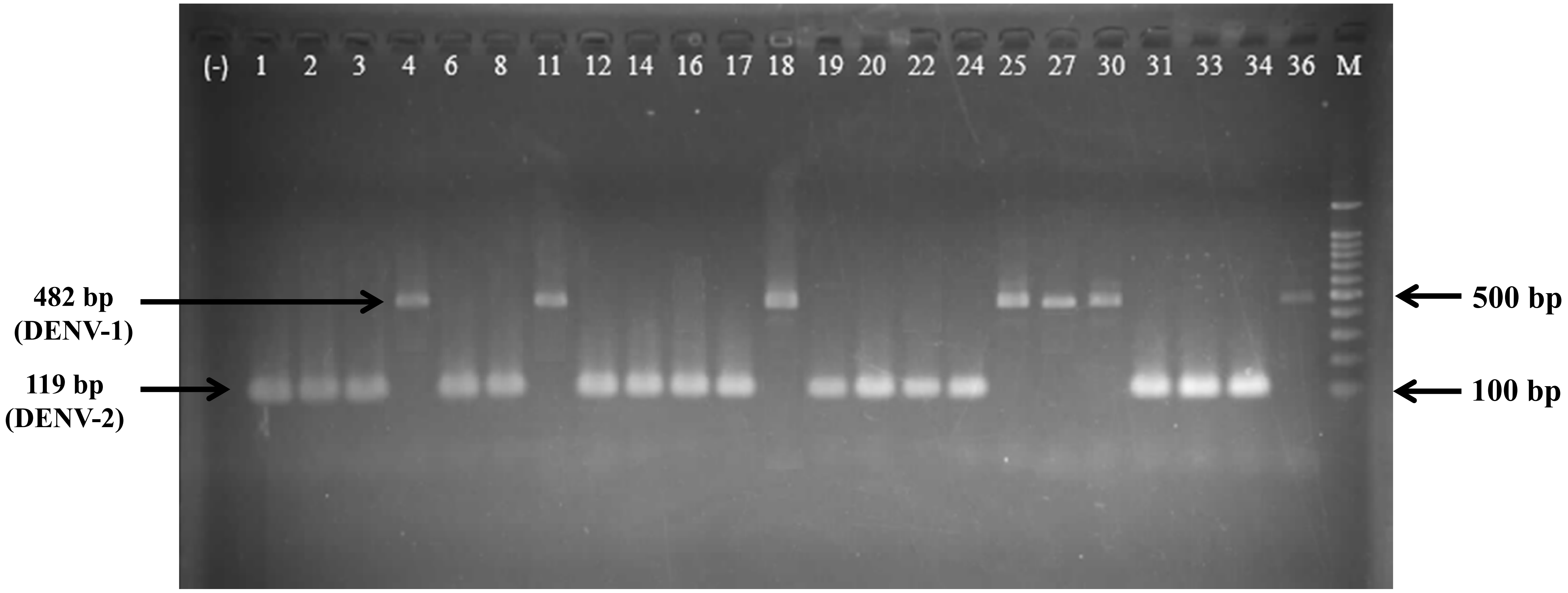

Figure S12: Typing of 70 dengue-positive clinical samples by nested RT-PCR

Numbers below wells indicate sample identities; (-), negative control; (M), Intron Sizer^{TM} -100 DNA Marker

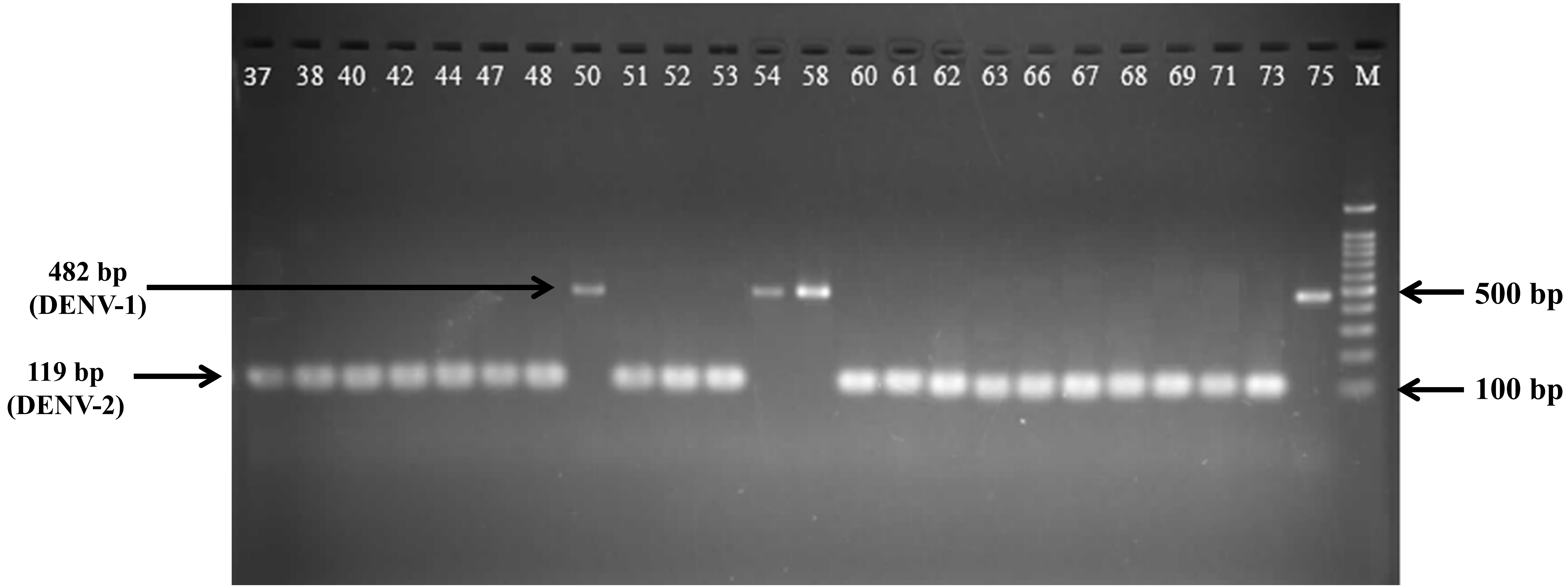

Figure S12 (continued): Typing of 70 dengue-positive clinical samples by nested RT-PCR
Numbers below wells indicate sample identities; (-), negative control; (M), Intron Sizer™ -100 DNA Marker

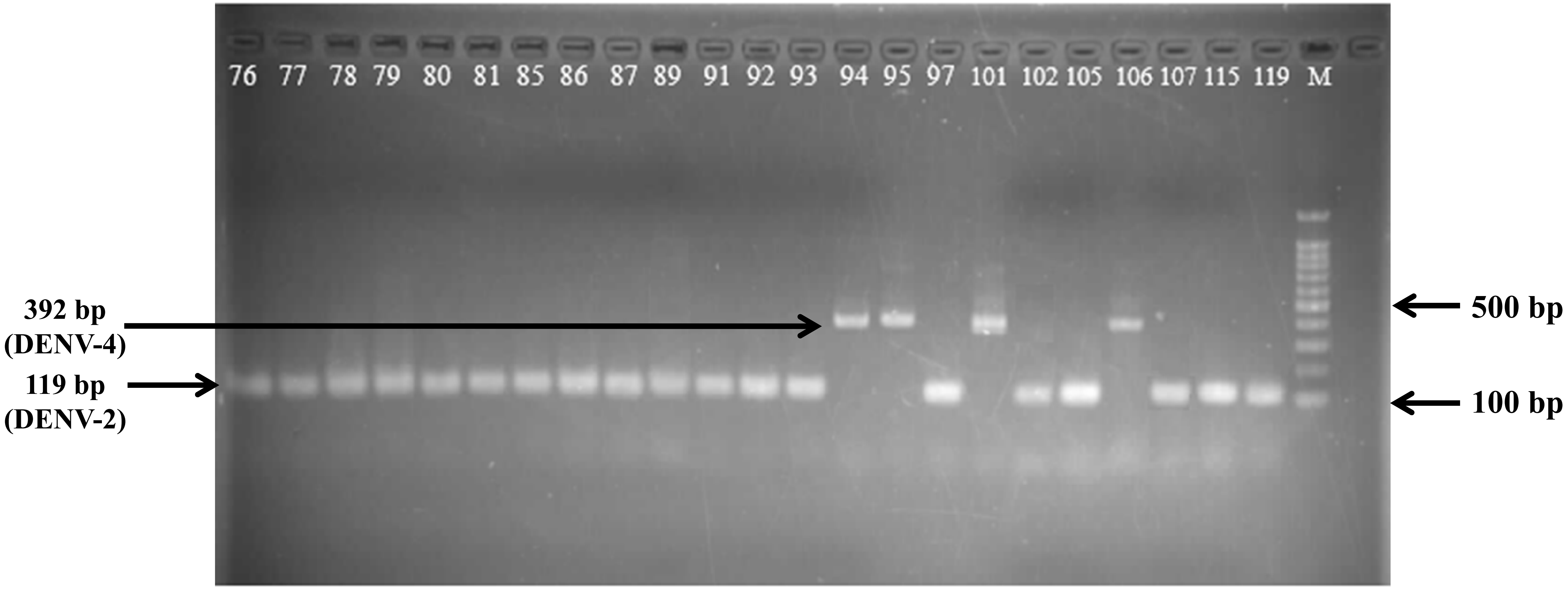

Figure S12 (continued): Typing of 70 dengue-positive clinical samples by nested RT-PCR
Numbers below wells indicate sample identities; (-), negative control; (M), Intron Sizer™ -100 DNA Marker

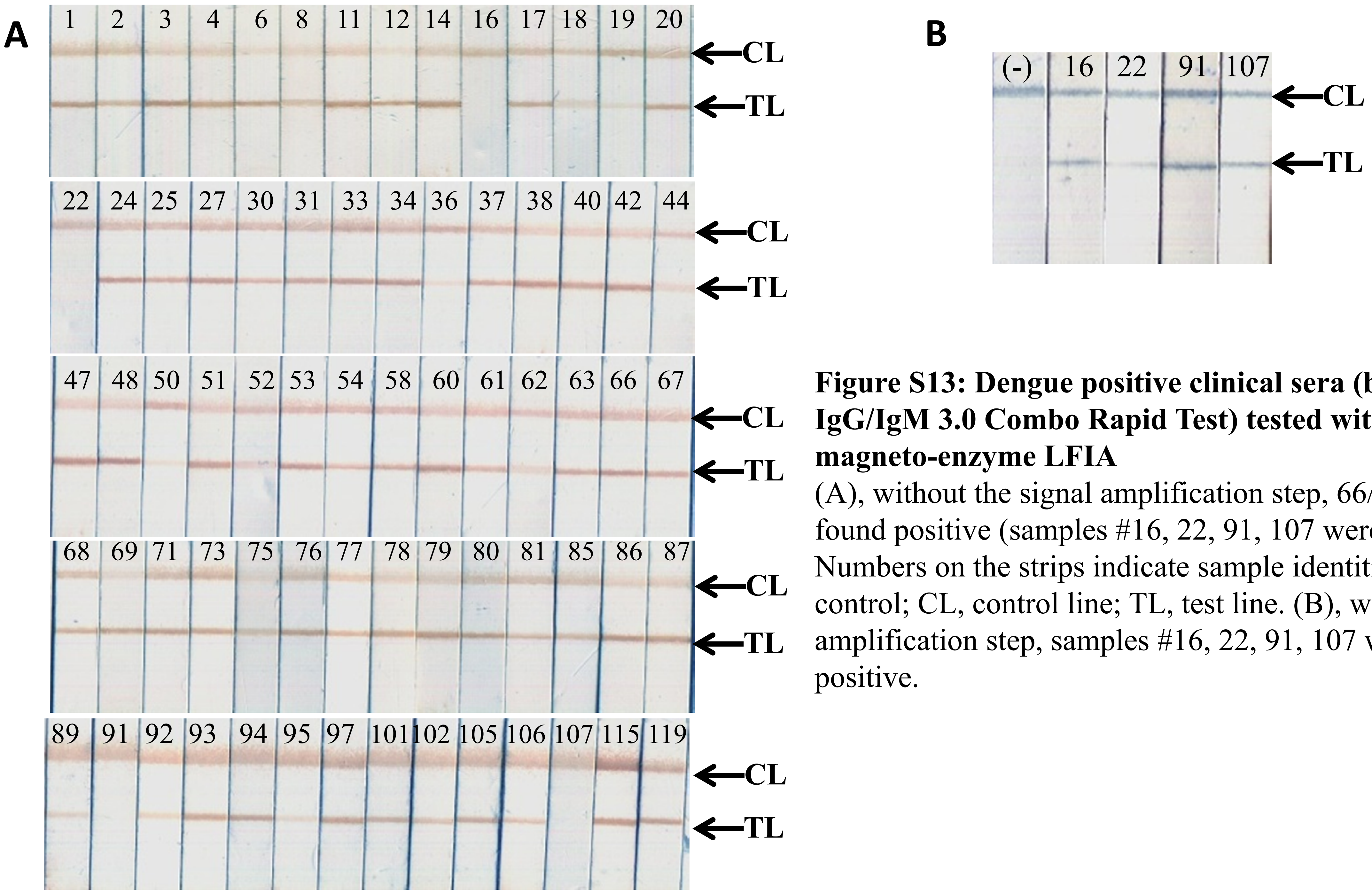

Figure S13: Dengue positive clinical sera (by RT-qPCR and IgG/IgM 3.0 Combo Rapid Test) tested with the magneto-enzyme LFIA

(A), without the signal amplification step, 66/70 samples were found positive (samples #16, 22, 91, 107 were found negative). Numbers on the strips indicate sample identities; (-), negative control; CL, control line; TL, test line. (B), with the signal amplification step, samples #16, 22, 91, 107 were found positive.
