# Supplementary material for: Development of a highly sensitive magneto-enzyme lateral flow immunoassay for dengue NS1 detection
Source: PeerJ. 2019 Sep 25;7:e7779. doi: 10.7717/peerj.7779 (PMC6765353; doi:10.7717/peerj.7779)
Supplement: Supplemental Information 6 [file peerj-07-7779-s006.pdf]

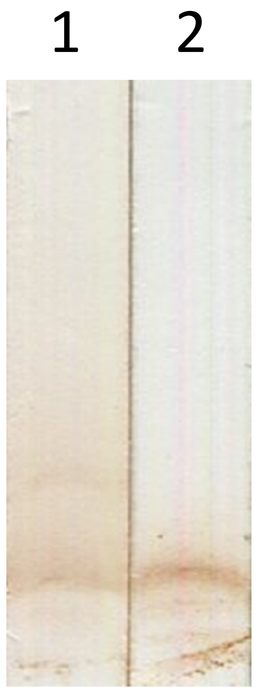

Additional Figure 1: Analysis of 300 nm Carboxyl-Adembeads on *CNPC-SS12 10 µ* membrane.
300 nm Carboxyl-Adembeads were flowed onto blank *CNPC-SS12 10 µ* membrane. (1), unconjugated beads. (2), antibody-conjugated beads.

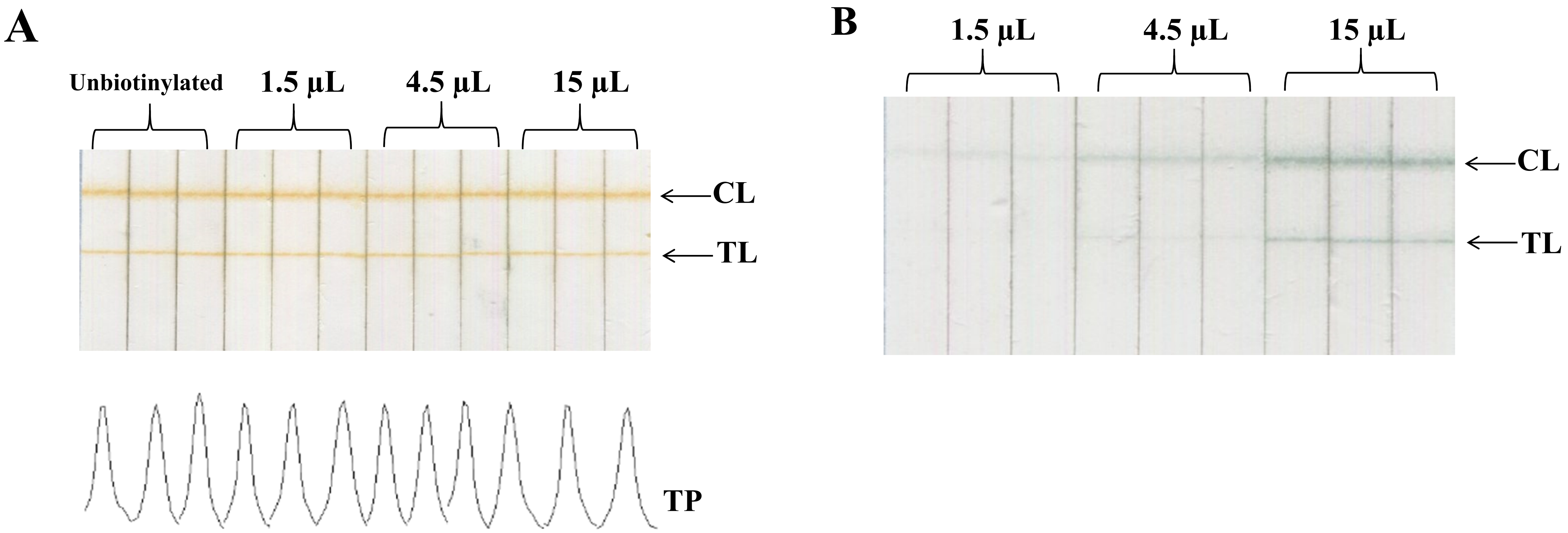

Additional Figure 2: Effects of biotinylation on reactivity of detection antibody and signal intensity after signal amplification step

NS1 antigen of dengue serotype 2 was immobilized on *CNPC-SS12 10 µ* nitrocellulose membrane at 1 µg/cm, followed by flowing of unbiotinylated or biotinylated antibody-conjugated magnetic nanoparticles. Biotinylation was performed using 1.5, 4.5 or 15 µL of 10 mM Sulfo-NHS-Biotin. The experiment was performed in 3 replicates. (A), reactivity of unbiotinylated and biotinylated antibody-conjugated magnetic nanoparticles with DENV-2 NS1. Numbers above the test strips indicate the amounts of Sulfo-NHS-Biotin used. CL, control line; TL, test line; TP, ImageJ plots of test line of corresponding strips. (B), effects of Sulfo-NHS-Biotin amounts on signal intensity after the signal amplification step. A total of 0.1 µg of detection conjugate was used for each test strip.

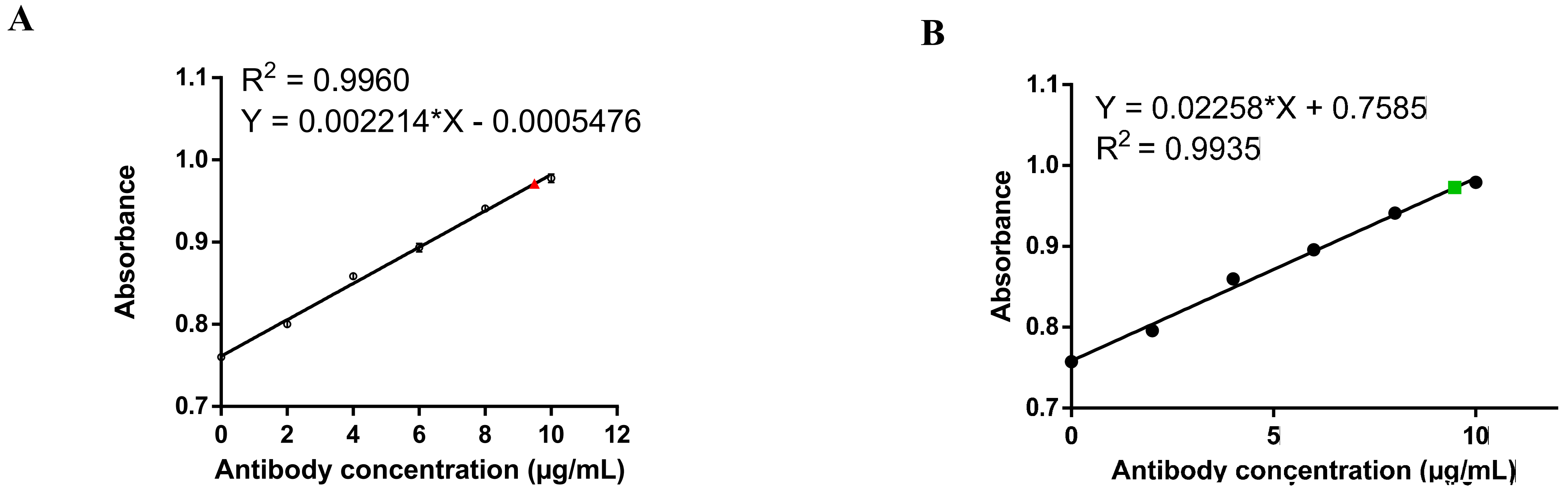

Additional Figure 3: Quantification of antibodies that were not conjugated to magnetic nanoparticles
After conjugation, the amount of unbound antibodies was quantified using a modified Bradford assay. The calibration curves are plotted using standard antibody solutions (black dots). The absorbances of the supernatants from antibody-magnetic nanoparticle conjugation are indicated by colored dots. (A), 10-2699 detection antibody from Fitzgerald. (B), HM164 detection antibody from EastCoast Bio
